# Proteomic Analysis of *Paracoccidioides brasiliensis* During Infection of Alveolar Macrophages Primed or Not by Interferon-Gamma

**DOI:** 10.3389/fmicb.2019.00096

**Published:** 2019-02-05

**Authors:** Edilânia Gomes Araújo Chaves, Juliana Alves Parente-Rocha, Lilian Cristiane Baeza, Danielle Silva Araújo, Clayton Luiz Borges, Milton Adriano Pelli de Oliveira, Célia Maria de Almeida Soares

**Affiliations:** ^1^Laboratório de Biologia Molecular, Instituto de Ciências Biológicas, Universidade Federal de Goiás, Goiânia, Brazil; ^2^Centro de Ciências Médicas e Farmacêuticas, Universidade Estadual do Oeste do Paraná, Cascavel, Brazil; ^3^Instituto de Patologia Tropical e Saúde Pública, Universidade Federal de Goiás, Goiânia, Brazil

**Keywords:** *Paracoccidioides* spp., proteome, metabolism, oxidative stress, alveolar macrophages, interferon gamma

## Abstract

Although members of the *Paracoccidioides* complex are not obligate intracellular pathogens, they present the ability to survive and multiply inside epithelial cells and phagocytes of mammals, which may favor the spread of the fungus in host tissues. Macrophages resident in the lung are the first line of defense against paracoccidioidomycosis (PCM), presenting mechanisms to control the pathogen dissemination through the granuloma formation or eliminating the fungus through phagocytosis. Phagocytosis triggers an oxidative burst, in which there is an increase in the production of toxic elements, derived from oxygen and nitrogen. The interior of the phagolysosome is a harsh environment to the internalized pathogens, since in addition to the oxygen and nitrogen reactive species, microorganisms face nutrient shortages and proteases activity. Through the NanoUPLC-MS^*E*^ technology, we analyzed the proteomic response of *Paracoccidioides brasiliensis* during the infection of alveolar macrophages primed or not by interferon gamma (IFN-γ). At 6 hs post-infection, only (IFN-γ)-primed macrophages were able to kill the fungus. We observed the regulation of amino acids degradation, tricarboxylic acid cycle, respiratory chain, ATP synthesis, glyoxylate cycle, as well as an increase in the expression of defense proteins related to oxidative stress, heat shock, and virulence factors under both conditions analyzed. However, some pathways described as essential for the survival of pathogens inside macrophages were observed only or with higher intensity in yeast cells recovered from non-primed macrophages, as phosphate pentoses pathway, methylcitrate cycle, synthesis of cell wall components, and mitochondrial activity. The data indicate that the intracellular environment of non-primed macrophages could be more permissive to the survival and multiplication of *P. brasiliensis*. The identification of key molecules for the establishment of infection can help the understanding of the nature of the parasite–host relationship and pathogenesis of PCM.

## Introduction

Paracoccidioidomycosis (PCM) is a systemic granulomatous mycosis caused by thermodimorphic fungi of the genus *Paracoccidioides*. This genus comprises five species, as following: *Paracoccidioides brasiliensis*, *Paracoccidioides americana*, *Paracoccidioides restrepiensis*, *Paracoccidioides venezuelensis*, and *Paracoccidioides lutzzi* (Matute et al., 2006; Carrero et al., 2008; Teixeira et al., 2009; Munoz et al., 2016; Turissini et al., 2017). PCM is an endemic disease of South America that currently infects at least 10 million people (Martinez, 2017; Turissini et al., 2017). The fungus mainly attacks the lungs, since the infection occurs through inhalation of infectious conidia or mycelia propagules. In few hours after contact with the pulmonary tissue, the fungus converts to the yeast phase, its parasitic form (McEwen et al., 1987). One of the main features of PCM is the formation of granulomas, since macrophages are one of the primary defense elements against *Paracoccidioides*. Macrophages form a giant multinucleated agglomerate capable of limiting the spread of yeast cells, besides eliminating the pathogens through phagocytosis (McEwen et al., 1987; Brummer et al., 1989; Fortes et al., 2011).

The intracellular environment of macrophages is a challenge for fungal survival. In addition to the low availability of nutrients and the action of proteases, there is an explosion of oxygen consumption during host–pathogen interaction (HPI), which has been associated with the microbicidal activity of phagocytes (Philippe et al., 2003; Fang, 2004; Haas, 2007). This reaction produces large amounts of the reactive oxygen species (ROS) and reactive nitrogen species (RNS). The oxidative and nitrosative stresses cause oxidation of proteins, lipids, and DNA which interferes in the replication of pathogens (Ferrari et al., 2011; Fang, 2004). Despite this, *Paracoccidioides* is able to survive and multiply inside phagocytes of mammals, which indicates that the fungus can subvert the phagocytic defenses to promote its spread through host tissues (Brummer et al., 1989; Moscardi-Bacchi et al., 1994; Hanna et al., 2000; Mendes-Giannini et al., 2004).

The intracellular survival of fungi has motivated several studies to investigate which are the strategies used by those pathogens to survive inside macrophages (Seider et al., 2010; Seider et al., 2014; Kasper et al., 2015; Erwig and Gow, 2016; Camacho and Nino-Vega, 2017). Proteomic and transcriptomic studies analyzed the metabolic adaptation of fungi to *in vitro* conditions of deprivation of carbon sources, mimicking the interior of the phagolysosome (Yin et al., 2004; Askew et al., 2009; Szilagyi et al., 2013; Lima et al., 2014; Baeza et al., 2017). Those studies show that fungi, including *Paracoccidioides*, present a metabolic reprogramming decreasing glycolysis and increasing alternative carbon pathways as glyconeogenesis, amino acid degradation, β-oxidation of fatty acids, and glyoxylate cycle. Corroborating transcriptional and proteomic data, it was observed that fungal strains presenting disrupted genes encoding proteins in those pathways have attenuated virulence during macrophage infection (Lorenz and Fink, 2002; Ramirez and Lorenz, 2007).

The success of mechanisms of intracellular evasion of pathogens depends on the activation profile of macrophages. Activation of phagocytes with INF-γ affects directly the viability of *Paracoccidioides* sp. cells interacting with macrophages, and is crucial to prevent the progression of disease. Infection assays performed with several members of the *Paracoccidioides* complex showed that the addition of INF-γ does not increase the phagocytosis index, but it confers microbicide activity to the macrophages in a dose-dependent manner (Brummer et al., 1989; Moscardi-Bacchi et al., 1994; Rodrigues et al., 2007). Moreover, deficiency in the immune system, related to low production of cytokines, have been associated with the evolution of PCM. Studies revealed that the production of IL-2 and INF-γ by peripheral blood mononuclear cells from patients affected by acute or chronic PCM was lower compared to healthy patients. INF-γ also plays an important role in the recruitment of defense cells to the lungs and for the efficiency of granulomas. Large amounts of IFN-γ were detected in compact granulomas of PCM-resistant mouse in relation to loose granulomas and multifocal lesions detected in susceptible animals (Souto et al., 2000, 2003; Bernard and Latge, 2001; Nishikaku et al., 2011).

To our knowledge, up-to-date, proteomic analysis of *Paracoccidioides* phagocytosed by macrophages with different activation patterns has not been performed. Previous studies from our group analyzed the proteomic response of *Paracccidioides* to macrophage interaction after 24 h and described modulation of energetic metabolism and of proteins related to stress response. The down-regulation of glycolysis and TCA cycle was observed and the up-regulation of ethanol production and fatty acid degradation occurs, suggesting the fungus preferentially uses anaerobic pathways to obtain energy. The up-regulation of proteins related to cell rescue and virulence, such as proteases and superoxide dismutases (SODs), are also described suggesting that this repertoire is required to *Paracoccidioides* survival inside macrophages (Parente-Rocha et al., 2015).

In this study, we compared the proteomic response of *P. brasiliensis*, *Pb*18 during interaction with alveolar macrophages, primed or not with INF-γ. Metabolic peculiarities between the two conditions, such as the activation of pentose-phosphate pathway, methylcitrate cycle, synthesis of cell wall components, and intense mitochondrial activity, were observed only in fungal cells recovered from non-primed macrophages. In both analyzed conditions, the fungus presented decrease of β-oxidation of fatty acids and protein synthesis, and increase in enzymes related to amino acid degradation, TCA and glyoxylate cycles, as alternative energy pathways, at 6 h post infection. The induction of proteins related to heat shock response, antioxidant response, and accumulation of virulence factors, were observed, predominantly in yeast cells after interaction with non-primed macrophages. While the primed cells showed fungicidal activity in the first 6 h of interaction, the interior of non-primed macrophages appears to be a favorable environment to the survival and multiplication of *P. brasiliensis.* When considering primed macrophages, the same metabolic pathways were induced comparing to the control, but at low levels than those observed in *P. brasiliensis* recovered from non-primed macrophages, suggesting that the primed macrophages difficult the fungus adaptation to survival. The identification of key molecules for the establishment of infection can help us to understand the nature of the parasite–host relationship and the factors that determine the evolution from asymptomatic infection to manifested disease.

## Materials and Methods

### Cultivation and Maintenance of Microorganism

*Paracoccidioides brasiliensis*, *Pb*18 (chronic PCM; São Paulo, Brazil; *P*. *brasiliensis*, *Pb*18) (Turissini et al., 2017) was used in all experiments. The yeast cells were maintained by sub culturing at 36°C in Fava Netto’s solid medium every 7 days (Fava-Netto, 1955). After this period, the cells were transferred to Fava Netto’s liquid medium for 72 h at 36°C under agitation at 150 rpm and used to perform the experiments.

### Cultivation and Maintenance of Alveolar Macrophages

The alveolar macrophages AMJ2-C11 (Rio de Janeiro Cell Bank – BCRJ/UFRJ, accession number 0039) are originated from *Mus musculus*. The cells were maintained in DMEM medium (Vitrocell Embriolife, Campinas, São Paulo, Brazil) containing bovine fetal serum 10% (v/v) at 36°C and 5% CO_2_. The culture medium was changed after the cells reach complete confluence.

### Macrophage Infection Assays

*Paracoccidioides brasiliensis*, *Pb*18 infection in AMJ2-C11 alveolar macrophages was performed in triplicates on 12-well polypropylene plates. In each well was plated a total of 10^6^ macrophages cells in DMEM medium containing or not IFN-γ (1 U/mL) (Sigma–Aldrich, St. Louis, MO, United States) and bovine fetal serum 10% (v/v). The plates were incubated for 12 h at 36°C and 5% CO_2_ until complete confluence. The medium was removed and a fresh DMEM medium containing or not IFN-γ (1 U/mL) and bovine fetal serum 10% (v/v) plus 5 × 10^6^ *Pb*18 cells were added to the macrophages. For all infection assays, the yeast cells obtained from a 72 h inoculum in Fava Netto’s liquid medium were passed through 0.70 μm-pore membrane filters, with the aid of syringe and needle. The plates were incubated at 36°C and 5% CO_2_ during 3, 6, 9, and 12 h. The wells were gently washed three times with sterile phosphate-buffered saline (PBS) and the macrophages were lysed by the addition of sterile water. The yeast cells were recovered by centrifugation at 8,000 × *g* for 10 min (Parente-Rocha et al., 2015). The pellet was diluted (1:100) and plated in solid brain heart infusion (BHI) medium, supplemented with bovine fetal serum 4% (v/v) and glucose 4% (v/v). Yeast cells viability was evaluated based on the number of colony-forming units (CFU), determined after growth at 36°C for 10 days. The control was obtained by incubating 5 × 10^6^ cells/mL in DMEM medium, added of bovine fetal serum 10% (v/v) for 6 h at 36°C and 5% CO_2_. Control fungal cells were washed with sterile water prior to protein extraction. Obtainment of adhered and internalized fungal cells indexes after interaction with primed and non-primed macrophages were performed by counting a total of 600 events (macrophages) in cover lips stained with Giemsa, as previously described (Gori and Scasso, 1994).

### Obtaining Protein Extracts

The proteomic analysis was carried out after 6 h post interaction of fungal and macrophage cells. Fungal protein extracts were obtained in biological triplicates from the three conditions to be analyzed: *Pb*18 cells recovered of primed macrophages (*Pb*18_P); *Pb*18 cells recovered of non-primed macrophages (*Pb*18_NP) and control cells (*Pb*18 cells not interacting with macrophages). The yeast cells were collected by centrifugation at 8,000 × *g* for 10 min and washed once with RapiGEST SF Surfactant 0.1% (v/v) (Waters Corporation, Billerica, MA, United States), followed by washing with ultrapure water and PBS 1×, in order to remove any contamination of the macrophage cells (Parente-Rocha et al., 2015; Pigosso et al., 2017). The pellet was ressuspended in extraction buffer (20 mM Tris–HCl, pH 8.8, and 2 mM CaCl_2_) and distributed in tubes containing glass beads (Sigma–Aldrich, St. Louis, MO, United States) in equal volume of the material. The suspension was processed on ice in BeadBeater equipment (BioSpec, Products Inc., Bartlesville, OK, United States) during three cycles of 30 s. The cell lysate was centrifuged at 10,000 × *g* during 15 min at 4°C and the protein content in the supernatant was quantified using the Bradford reagent (Sigma–Aldrich, St. Louis, MO, United States) using bovine serum albumin (BSA) as standard.

### Protein Digestion for NanoUPLC-MS^E^ Analysis

Proteins were enzymatically digested with tripsin as described previously, with some modifications (Murad et al., 2011). Briefly, a total of 150 μg of protein (previous item) of each sample was added to 10 μL of 50 mM ammonium bicarbonate, pH 8.5, in a microcentrifuge tube. Next, 75 μL of RapiGEST^TM^ SF Surfactante (0.2% v/v) (Waters Corporation, Billerica, MA, United States) was added and the sample was vortexed and incubated in a dry bath at 80°C for 15 min. In each sample were added 2.5 μL of 100 mM dithiothreitol (GE Healthcare, Piscataway, NJ, United States), at 60°C for 30 min, while cysteines were alkylated by the addition of 2.5 μL of 300 mM iodoacetamide (GE Healthcare, Piscataway, NJ, United States) for 30 min, at room temperature in the dark. The digestion of proteins was performed by the addition of 30 μL of trypsin 0.05 μg/μL (Promega, Madison, WI, United States) at 37°C, in dry bath, for 16 h. Then, 30 μL of trifluoroacetic acid (TFA) solution 5% (v/v) was added to the samples, followed by incubation for 90 min at 37°C, for digestion stop, and precipitation of the RapiGEST reagent. The samples were centrifuged at 18,000 × *g* for 30 min and the supernatant was transferred to a new tube and dried in a speed vacuum (Eppendorf, Hamburg, Germany) for 2 h. The pellet containing peptides was suspended in 80 μL of a solution containing 20 mM of ammonium formiate and 150 fmol/μL of PHB (Rabbit Phosphorylase B) (Waters Corporation, Billerica, MA, United States) (MassPREP protein), as internal standard. The tryptic peptides were analyzed using a nanoACQUITY UPLC^®^ M-Class system (Waters Corporation, Billerica, MA, United States) coupled to Synapt G1 MS^TM^ mass spectrometer (Waters Corporation, Billerica, MA, United States), equipped with a NanoElectronSpray source and two mass analyzers: a quadrupole and a time-of-flight (TOF) operating in the V-mode. Nanoscale LC separation of tryptic peptides was performed with two reverse phase columns. The peptides were separated using a gradient of 11.4, 14.7, 17.4, 20.7, and 50% (v/v) of acetonitrile/0.1% (v/v) formic acid, with a flow rate of 2.000 μL/min. Data were obtained using the instrument in the MS^E^ mode, which switches the low energy (6 V) and elevated energy (40 V) acquisition modes every 0.4 s. The lock mass was used for calibration of the apparatus, using a constant flow rate of 0.2 μL/min at concentration of 200 fmol protein GFP [Glu]^1^-Fibrinopeptide B human (*m/z* 785.8426) (Sigma–Aldrich, St. Louis, MO, United States). The samples were analyzed in triplicate, from three independent experiments.

### Data Processing and Protein Identification

Mass spectrometry raw data of peptide fractions were processed using the ProteinLynx Global SERVER (PLGS) platform (Waters Corporation, Billerica, MA, United States). Then, the processed spectra were searched against *P. brasiliensis*, *Pb*18 protein sequences together with reverse sequences. The mass error tolerance for peptide identification was under 50 ppm. Protein identification criteria were as following: detection of at least two fragment ions per peptide; five fragment ions per protein; the determination of at least one peptide per protein; carbamidomethylation of cysteine as a fixed modification; phosphorylation of serine, threonine, and tyrosine; and oxidation of methionine were considered as variable modifications; maximum protein mass (600 kDa); one missed cleavage site was allowed for trypsin; maximum false positive ratio (FDR) of 5% was allowed. For the analysis of the level of protein quantification, the observed intensity measures were normalized using a protein that showed lower coefficient of variance between the different conditions analyzed and present in all replicates. Expression^E^ informatics v.3.0.2 was used for proper quantitative comparisons. The identified proteins were organized by the expression algorithm, into a statistically significant list, corresponding to induced and reduced regulation ratios between the different conditions analyzed. The mathematical model used to calculate the ratios was a part of the Expression algorithm inside the PLGS software (Waters Corporation, Billerica, MA, United States) (Geromanos et al., 2009). The minimum repeat rate for each protein in all replicates (nine in total for each condition) was 6. Proteins that presented 40% of differences in expression values, when compared among the different conditions, were considered regulated. Tables of peptides and proteins generated by the PLGS were merged, and the data of dynamic range, peptide detection type, and mass accuracy were calculated for each sample, as previously described using the software MassPivot v1.0.1 (Murad and Rech, 2012), FBAT (Lange et al., 2004), Spotfire^®^ v8.0 program (TIBCO software), and Microsoft Excel^®^ (Microsoft) was used for table manipulations. Uniprot^1^ and Pedant on MIPS^2^ database were used for functional classification. NCBI database was employed for annotation of uncharacterized proteins^3^. The heat maps were performed by using MultiExperiment Viewer tool version 4.9^4^.

### Analysis of Cell Wall Components by Fluorescence Microscopy

To evaluate the cell wall components glucans, glycosylated proteins, and chitin contents, the yeast cells recovered of macrophages were stained with aniline blue (AB) (Sigma Aldrich, Missouri, United States) for 5 min, concanavalin A (ConA) TYPE VI conjugated to FITC 100 μg/mL (Sigma–Aldrich, St. Louis, MO, United States) for 30 min and calcofluor white (CFW) 100 μg/mL (Sigma–Aldrich, St. Louis, MO, United States) for 30 min (de Curcio et al., 2017). These experiments were performed independently for each substance. Stained samples were visualized under a fluorescence microscope (Zeiss Axiocam MRc-Scope A1, Oberkochen, Germany). Images were obtained at bright field, at 340–380 nm for AB, 470–480 nm for Con A, and at 395–440 nm for CFW. A minimum of 100 cells on each microscope slide were used to evaluate fluorescence intensity in triplicates. The AxioVision Software (Carl Zeiss AG, Germany) determined the fluorescence intensity (in pixels) and standard error of each analysis. Statistical comparisons were performed using the Student’s *t*-test and *p* ≤ 0.05 was considered statistically significant.

### Evaluation of Mitochondrial Activity

*Paracoccidioides brasiliensis*, *Pb*18 yeast cells recovered from macrophages and control cells (as previously described) were centrifuged at 8,000 × g for 10 min. The pellet was diluted in PBS 1× to a concentration of 1 × 10^6^ cells/mL. To label mitochondria, the cells were stained with Mitotracker Green FM (400 nM; Molecular Probes, M7514) for 45 min at 37°C. Then, the cells were washed three times with PBS 1× and labeled with rodhamine (2.4 μM) for 45 min at 37°C, according to the manufacturer’s protocol (Invitrogen, Carlsbad, CA, United States) and washed three times with PBS 1×. Labeled cells were observed under a fluorescence microscope (Zeiss Axiocam MRc-Scope A1, Oberkochen, Germany) and photographed at bright field, and at 450–490 nm for the Mitotracker and 546–512 nm for rosamine dye probes. A minimum of 100 cells on each microscope slide were used to evaluate fluorescence intensity in triplicate. The AxioVision Software (Carl Zeiss AG, Germany) determined the fluorescence intensity (in pixels) and standard error of each analysis. Statistical comparisons were performed using the Student’s *t*-test and *p* ≤ 0.05 was considered statistically significant.

## Results

### *P. brasiliensis* Survival Into Alveolar Macrophages

**FIGURE 1:**
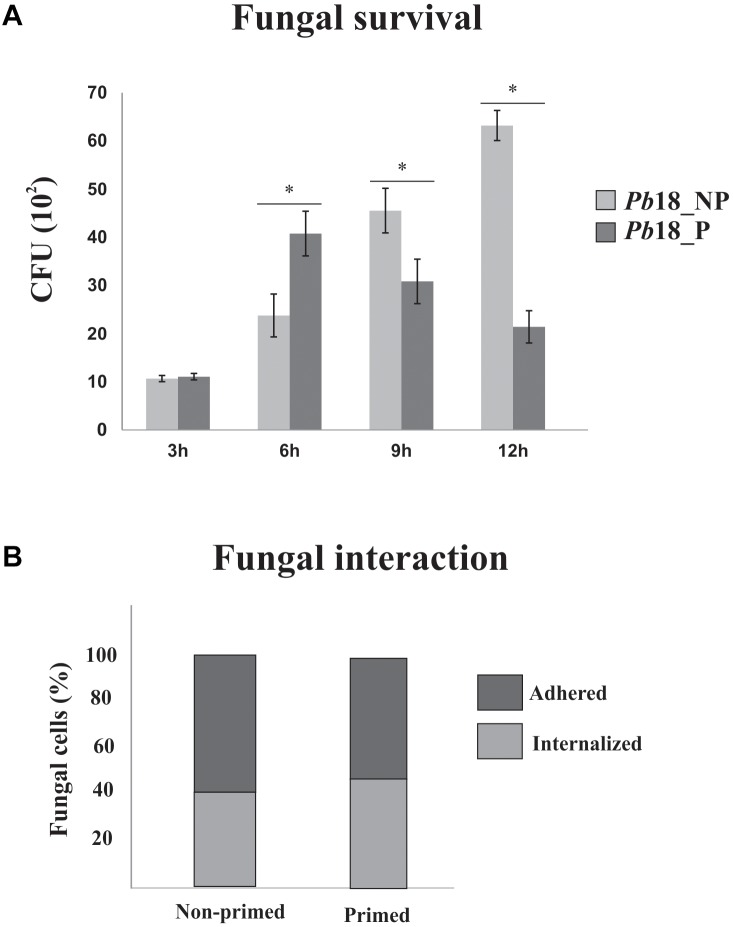
*P. brasiliensis*, *Pb*18 infection in alveolar macrophages. **(A)** Recovery of *P. brasiliensis* fungal cells after interaction with primed and non-primed macrophages. *P. brasiliensis*, *Pb*18 was recovered from AMJ2-C11 primed (*Pb*18_P) and non-primed (*Pb*18_NP) alveolar macrophages, by INF-γ after: 3, 6, 9, and 12 h of interaction. The viable yeast cells recovered from macrophages were determined based on the number of CFUs. The error bars indicate the mean ± standard deviation of the values obtained from the triplicate of independent experiments. ^∗^, Significantly different results; *P*-value ≤ 0.05. **(B)** Percentage of adhered and internalized fungal cells after interaction with primed and non-primed macrophages. The interaction assay was performed in glass slides; fungi cells were washed, fixed, and stained with Giemsa. A total of 600 macrophages were counted.

*Paracoccidioides brasiliensis*, *Pb*18 infection in AMJ2-C11 alveolar macrophages was performed to determine the best time of infection, with higher rate of internalization/adhrence of viable fungi cells. Times of 3, 6, 9, and 12 h of infection were analyzed regarding the number of CFUs in *Pb*18 yeast cells recovered of primed macrophages (*Pb*18_P) and non-primed macrophages (*Pb*18_NP). Figure 1A shows that the number of viable fungal cells increases inside the primed macrophage until 6 h. After this time, the number of viable fungi decreases in primed macrophages, suggesting killing of yeast cells by the macrophages after 9 h of interaction. In this way, the proteomic response of *P. brasiliensis*, after 6 h of interaction with macrophages, was investigated to analyze the differences in the initial proteomic response of the fungus during interaction with macrophages with different activation patterns. In order to evaluate the index of fungal cells adhered/internalized cover lips stained by Giemsa were analyzed after 6 h of interaction of fungal cells with primed and non-primed macrophages. The results are shown in Figure 1B. After interaction with non-primed macrophages, 42% of fungal cells interacting with macrophages are internalized and 58% are adhered to macrophages. A slight increase in the internalization was observed after interaction of fungal cells with primed macrophages (48%) while the percentage of adhered cells index was 52% after interaction of fungal cells with primed macrophages.

### Proteomic Data Quality Analysis

Protein extracts were obtained in biological triplicates. Protein quantification was performed using Nano-UPLCMS^E^ and the protein and peptide data were generated by the PLGS (Supplementary Figure 1). The false positive rates of proteins obtained from *Pb*18_P were 3.80%, *Pb*18_NP 5.88%, and control 2.99%. Those experiments resulted in 53,745; 47,941; and 61,561 identified peptides, from *Pb*18_P; *Pb*18_NP; and control, respectively. The values of 50.1, 49.2, and 53.9% were obtained from peptide match type data in the first pass and 7.5, 7.6, and 9.7% from the second pass, for *Pb*18_P, *Pb*18_NP, and control, respectively. The percentages of 16.2, 16.5, and 14% of total peptides were identified by a missed trypsin cleavage, whereas an in-source fragmentation was 11, 11.2, and 11.8% for *Pb*18_P, *Pb*18_NP, and control, respectively. The peptides identification within the first and second pass (PepFrag 1 and 2) were predominantly higher than 56%, and source fragmentation and missed cleavage values did not exceed 20% in all analyzed conditions (Supplementary Figure 1A; Murad and Rech, 2012). The peptide parts per million errors (ppm) indicated that the majority, 82, 83.9, and 79.3% of identified peptides, were detected with an error of less than 10 ppm for *Pb*18_P, *Pb*18_NP, and control, respectively (Supplementary Figure 1B). The obtained results from dynamic range detection indicated that a 3-log range concentration and a good detection distribution of high and low molecular weights were obtained in all analyzed conditions (Supplementary Figure 1C).

Five hundreds and thirty eight proteins were detected, considering the proteomic analysis. The description of all detected proteins is shown in Supplementary Table 1. Supplementary Figure 2 ilustrates this data. Among proteins identified during interaction of fungal cells with primed macrophages, 199 proteins were detected in similar amounts and were considered not regulated proteins. A total of 135 proteins were up-regulated in fungal cells after interaction with primed macrophages. Proteins detected only in yeast cells recovered from primed macrophages, in number of 54 proteins were also considered up-regulated. The down regulated proteins include those detected in higher amounts in the control fungal cells (67 proteins) and proteins detected only in the control condition in number of 34. The data obtained from fungal cells after interaction with non-primed macrophages include: 140 proteins that were detected in similar amounts in control fungal cells and in yeast cells after interaction with non-primed macrophages, 222 proteins up-regulated in the fungal cells after interaction with non-primed macrophages, and 54 proteins detected only after interaction with primed macrophages. Concerning to down regulated proteins, 15 were detected in higher amounts in control fungal cells and 49 proteins were exclusively detected in control fungal cells.

### Regulated Proteins in *P. brasiliensis* During Infection of Primed Macrophages

**FIGURE 2:**
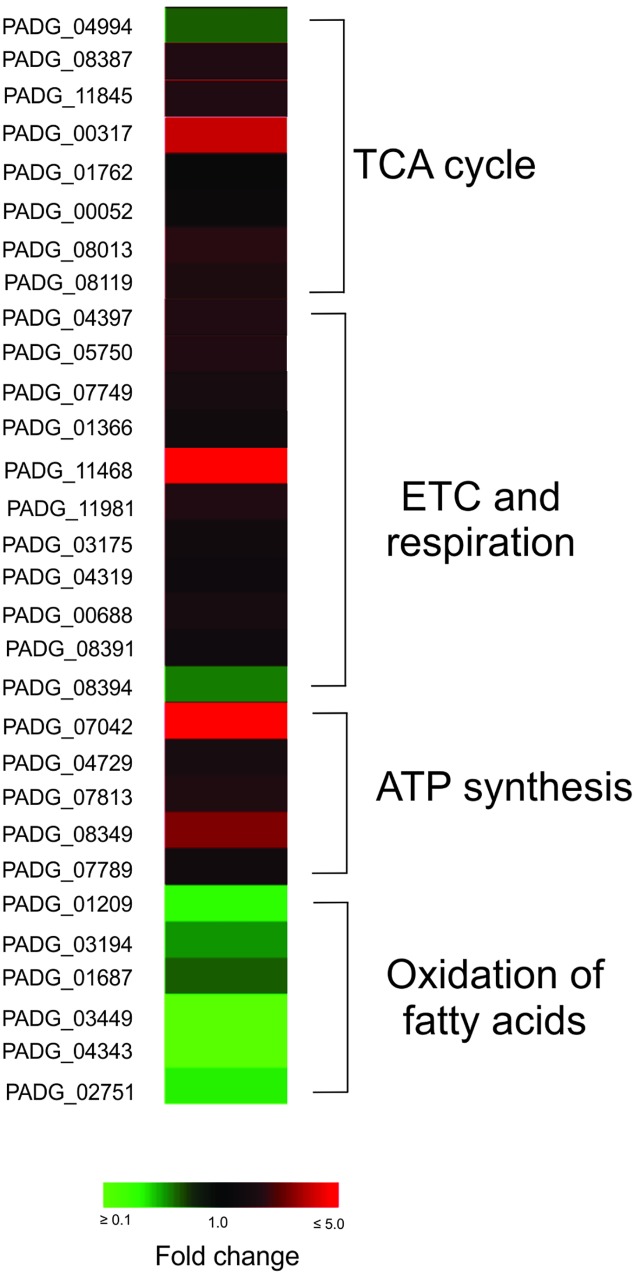
Heat map showing up and down regulated cellular processes in *P. brasiliensis* infecting IFNγ primed macrophages. Tricarboxylic acid cycle (TCA), electron transport chain (ETC), and ATP synthesis were predominantly up-regulated and oxidation of fatty acids is predominantly down-regulated in *P. brasiliensis* during incubation in IFNγ primed macrophages. TCA, tricarboxylic cycle acid; ETC, electron transport chain; PADG_04994, citrate synthase subunit 1; PADG_08387, citrate synthase, mitochondrial; PADG_11845, aconitate hydratase, mitochondrial; PADG_00317, succinyl-CoA ligase subunit beta; PADG_01762, oxoglutarate dehydrogenase (succinyl-transferring), E1 component; PADG_00052, succinate dehydrogenase [ubiquinone] flavoprotein subunit, mitochondrial; PADG_08013, succinate dehydrogenase [ubiquinone] iron-sulfur subunit, mitochondrial; PADG_08119, fumarate hydratase, mitochondrial; PADG_04397, cytochrome c oxidase subunit 4, mitochondrial; PADG_05750, putative cytochrome c oxidase subunit; PADG_07749, NAD(P)H:quinone oxidoreductase, type IV; PADG_01366, NADH-ubiquinone oxidoreductase; PADG_11468, electron transfer flavoprotein beta-subunit; PADG_11981, V-type proton ATPase catalytic subunit A; PADG_03175, V-type proton ATPase subunit F; PADG_04319, V-type ATPase, G subunit; PADG_00688, F-type H+-transporting ATPase subunit H; PADG_08391, plasma membrane ATPase; PADG_08394, cytochrome b–c1 complex subunit 2; PADG_07042, ATP synthase F1, delta subunit; PADG_04729, ATP synthase subunit D, mitochondrial; PADG_07813, ATP synthase F1, gamma subunit; PADG_08349, ATP synthase subunit beta, mitochondrial; PADG_07789, ATP synthase subunit delta, mitochondrial; PADG_01209, enoyl-CoA hydratase; PADG_03194, 3-ketoacyl-CoA thiolase B; PADG_01687, 3-ketoacyl-CoA thiolase; PADG_03449, isopentenyl-diphosphate delta-isomerase; PADG_04343, short chain dehydrogenase/reductase; PADG_02751, acetyl-CoA acetyltransferase.

Protein extracts of *P. brasiliensis*, *Pb*18 recovered of primed macrophages were compared with control samples by using MS^E^ technology. A total of 290 regulated proteins was detected in *Pb*18_P; from those 189 were up-regulated proteins and 101 down-regulated proteins (Supplementary Table 2). Supplementary Table 1 shows that after 6 h of interaction with primed macrophages, *P. brasiliensis* presents accumulation of proteins and enzymes from the TCA cycle, electron transport chain, and ATP synthesis, proteins related to oxidative stress protection, such as thioredoxin reductase (TrxR) (accession number PADG_01551) and SOD (accession number PADG_07418), and virulence factors such as serine proteinase (PADG_07422). Also, potential transcription factors are accumulated. In counterpart, down-regulation of proteins related with the β-oxidation of fatty acids was described. The regulation of proteins related to the tricarboxylic acid cycle, electron transport chain, ATP synthesis, and oxidation of fatty acids is visualized in the heat map presented in Figure 2.

### Proteins Regulated in *P. brasiliensis* During Infection of Non-primed Macrophages

A total of 340 regulated proteins were detected in *Pb*18 yeast cells recovered from macrophages not incubated with IFN-γ. From the total, 276 were up-regulated proteins and 64 were down-regulated (Supplementary Table 3). *P. brasiliensis*, *Pb*18 cells recovered from macrophages not incubated with IFN-γ showed an increase of proteins related to pentose-phosphate pathway, TCA, methylcitrate cycle, electron transport, and ATP synthesis, as well as transcription factors. Molecules related to cell rescue, defense, and virulence are also particularly accumulated. Enzymes of carbohydrate metabolism, related to the synthesis of cell wall precursors, were also induced. The negative regulation of enzymes from beta-oxidation of fatty acids was also described (Supplementary Table 3).

### Comparative Analysis of the Proteome of *P. brasiliensis* Recovered From IFN-γ Primed and Non-primed Macrophages

**FIGURE 3:**
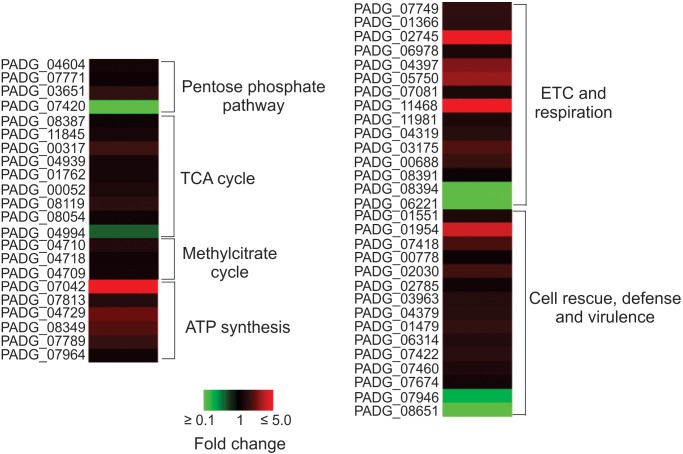
Heat map showing the cellular processes predominantly up regulated in non-primed macrophages. The most up-regulated processes are ETC and ATP synthesis. TCA, tricarboxylic cicle acid; ETC, electron transport chain; PADG_04604, transketolase; PADG_07771, 6-phosphogluconolactonase; PADG_03651, 6-phosphogluconate dehydrogenase, decarboxylating 1; PADG_07420, transaldolase; PADG_08387, citrate synthase mitochondrial; PADG_11845, aconitate hydratase mitochondrial; PADG_00317, succinyl-CoA ligase subunit beta; PADG_04939, succinyl-CoA, 3-ketoacid-coenzyme A transferase; PADG_01762, oxoglutarate dehydrogenase; PADG_00052, succinate dehydrogenase [ubiquinone] flavoprotein, mitochondrial; PADG_08119, fumarate hydratase, mitochondrial; PADG_08054, malate dehydrogenase, NAD-dependent; PADG_04994, ATP-citrate-lyase; PADG_04710, 2-methylcitrate synthase, mitochondrial; PADG_04718, 2-methylcitrate dehydratase; PADG_04709, methyl-isocitrate lyase; PADG_07042, ATP synthase F1, delta subunit; PADG_07813, ATP synthase F1, gamma subunit; PADG_04729, ATP synthase subunit D, mitochondrial; PADG_08349, ATP synthase subunit beta, mitochondrial; PADG_07789, ATP synthase subunit delta, mitochondrial; PADG_07964, vacuolar ATP synthase subunit E; PADG_07749, NAD(P)H:quinone oxidoreductase, type IV; PADG_01366, NADH-ubiquinone oxidoreductase 1 alpha subcomplex subunit 5; PADG_02745, NADH-ubiquinone oxidoreductase Fe–S protein 6; PADG_06978, cytochrome C; PADG_04397, cytochrome c oxidase subunit 4, mitochondrial; PADG_05750, cytochrome c oxidase subunit; PADG_07081, electron transfer flavoprotein subunit alpha; PADG_11468, electron transfer flavoprotein beta-subunit; PADG_11981, V-type proton ATPase catalytic subunit A; PADG_04319, V-type ATPase, G subunit; PADG_03175, V-type proton ATPase subunit F; PADG_00688, F-type H+-transporting ATPase subunit H; PADG_08391, plasma membrane ATPase; PADG_08394, cytochrome b–c1 complex subunit 2; PADG_06221, formate dehydrogenase; PADG_01551, thioredoxin reductase; PADG_01954, superoxide dismutase 2 Fe–Mn; PADG_07418, superoxide dismutase 1 Cu–Zn; PADG_00778, Hsp70; PADG_02030, Hsp90 co-chaperone Cdc37; PADG_02785, heat shock protein Hsp88; PADG_03963, 30 kDa heat shock protein; PADG_04379, heat shock protein STI1; PADG_01479, γ-glutamyltransferase; PADG_06314, carboxypeptidase Y; PADG_07422, serine proteinase; PADG_07460, vacuolar aminopeptidase; PADG_07674, carbonic anhydrase; PADG_07946, peroxisomal matrix protein; PADG_08651, peroxisomal hydratase–dehydrogenase–epimerase.

Yeast cells interacting with non-primed macrophages depicted faster metabolic adaptation, activating alternative routes such as methyl citrate cycle, in addition to amino acid degradation, pentose-phosphate pathway, electron transport chain, and ATP synthesis, as depicted in Figure 3. These data indicate that the intracellular environment of primed macrophages presents barriers that may hinder the adaptation of the fungus to the cell environment. Corroborating this hypothesis, *Pb*18_NP cells present a higher number of proteins and abundance of enzymes related to response to oxidative stress and virulence factors, as shown in Table 1 and Figure 3. SOD2, for example, had its expression more than twice higher in *Pb*18_NP compared to *Pb*18_P.

**Table 1:** Up-regulated proteins putatively related to cell rescue, defense, and virulence in *Pb*18_A and *Pb*18_NA.

Accession number^1^	Protein description	*Pb*18_P^2^	*Pb*18_NP^2^
**Defense and virulence**			
PADG_01479	Gamma-glutamyltransferase	1.76	2.46
PADG_06314	Carboxypeptidase Y	ND	1.97
PADG_07460	Vacuolar aminopeptidase	1.53	1.82
PADG_07422	Serine proteinase	1.8	2.17
PADG_07674	Carbonic anhydrase	ND	1.55
**Stress response**			
PADG_00778	Hsp70	ND	1.43
PADG_02030	Hsp90 co-chaperone Cdc37	2..22	2.71
PADG_02785	Heat shock protein Hsp88	1.5	1.48
PADG_03963	30 kDa heat shock protein	1.84	2.05
PADG_04379	Heat shock protein STI1	1.99	2.09
**Detoxification**			
PADG_01551	Thioredoxin reductase	1.56	1.84
PADG_01954	Superoxide dismutase 2 Fe–Mn, mitochondrial	1.99	4.72
PADG_07418	Superoxide dismutase 1 Cu–Zn, cytosolic	1.99	2.78

*^1^Accession number obtained in the Paracoccidioides database available at http://www.broadinstitute.org/annotation/genome/paracoccidioides_brasiliensis/MultiHome.html. ^2^Ratio values were obtained by dividing the values of protein abundance (in fmol) from Pb18 during infection of activated and non-activated macrophages, by the abundance in control. Proteins with a minimum fold change of 40% were considered regulated. ND, non-detected proteins.*

### *P. brasiliensis*, *Pb*18 Is Apparently Synthesizing More Cell Wall Components in Non-primed Macrophages

**FIGURE 4:**
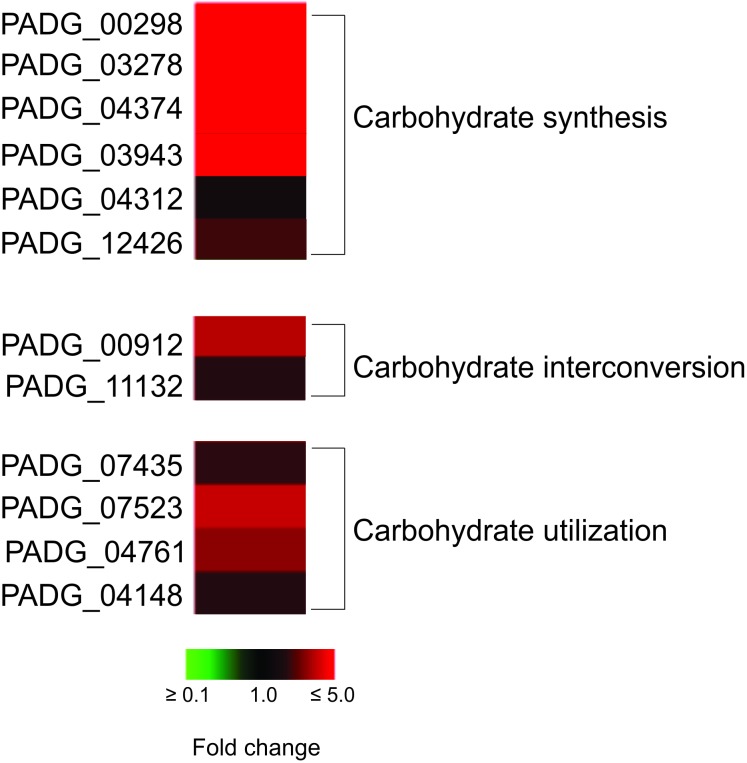
Heat map of proteins related to carbohydrate synthesis, interconversion, and utilization. The proteins related to these processes are predominalty up-regulated in *P. brasiliensis* during interaction with non-primed macrophages. PADG_00298, FGGY-family carbohydrate kinase; PADG_03278, inositol-3-phosphate synthase; PADG_04374, UTP-glucose-1-phosphate uridylyltransferase; PADG_03943, phosphomannomutase; PADG_04312, UDP-*N*-acetylglucosamine pyrophosphorylase; PADG_12426, 1,4-alpha-glucan-branching enzyme; PADG_00912, UDP-galactopyranose mutase; PADG_11132, phosphoglucomutase; PADG_07435, sorbitol utilization protein SOU2; PADG_07523, neutral alpha-glucosidase AB; PADG_04761, mannosyl-oligosaccharide glucosidase; PADG_04148, alpha-mannosidase.

We observed an increase of enzymes that participate in the synthesis of precursors of cell wall components as chitin, glycan, glycoproteins, and glycosylated compounds, mainly in *Pb*18_NP (Table 2; Figure 4). Enzymes as neutral alpha-glucosidase AB, mannosil-oligosaccharide glucosidase, and alpha-mannosidase are involved in glycoprotein biosynthesis through the *N*-glycosylation process (Almeida et al., 2016). UDP-galactopyranose mutase, UTP-glucose-1-phosphate uridylyltransferase, and UDP-*N*-acetylglucosamine pyrophosphorylase form UDP-sugars, substrates used in hundreds of glycosylation reactions (e.g., for protein and lipid glycosylation, synthesis of sucrose, and cell wall polysaccharides). UDP-*N*-acetylglucosamine pyrophosphorylase generates UDP-*N*-acetyl-D-glucosamine, which is used in the synthesis of chitin, for example (Decker et al., 2017). The up-regulation of the proteins involved in carbohydrate synthesis, interconversion, and utilization is shown in Figure 4. These data indicate that *P. brasiliensis* may be synthesizing components of the cell wall during infection, especially within non-primed macrophages.

**Table 2:** Up-regulated proteins putatively related to the homeostasis of cell wall componets in *Pb*18_A and *Pb*18_NA.

Accession number^1^	Protein description	*Pb*18_P^2^	*Pb*18_NP^2^
***N*-glycosylation**			
PADG_07523	Neutral alpha-glucosidase AB	1.91	2.54
PADG_04761	Mannosyl-oligosaccharide glucosidase	1.84	2.25
PADG_04148	Alpha-mannosidase	ND	1.7
**UDP-sugars**			
PADG_00912	UDP-galactopyranose mutase	ND	2.46
PADG_04374	UTP-glucose-1-phosphate uridylyltransferase	^∗^	*^∗^*
PADG_04312	UDP-*N*-acetylglucosamine pyrophosphorylase	ND	1.4
**Mannan**			
PADG_03943	Phosphomannomutase	^∗^	*^∗^*

*^1^Accession number obtained in the Paracoccidioides database available at http://www.broadinstitute.org/annotation/genome/paracoccidioides_brasiliensis/MultiHome.html. ^2^Ratio values were obtained by dividing the values of protein abundance (in fmol) from Pb18 during infection of activated and non-activated macrophages, by the abundance in control. Proteins with a minimum fold change of 40% were considered regulated. ^∗^Proteins detected in P. brasiliensis Pb18 only during respective macrophage infection. ND, non-detected proteins.*

**FIGURE 5:**
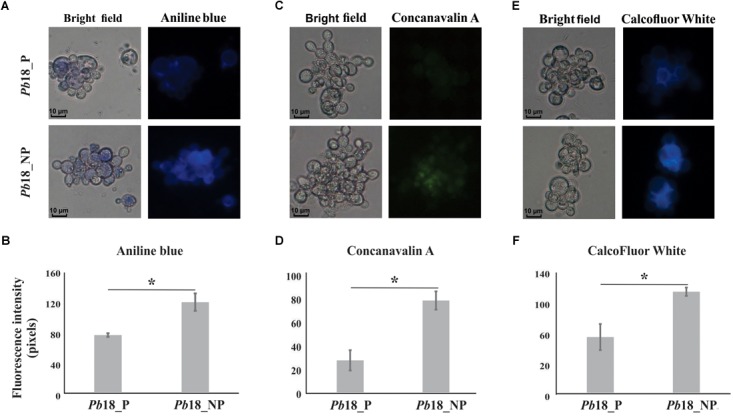
Evaluation of cell wall components of *P. brasiliensis Pb*18 recovered from alveolar macrophages. The yeast cells recovered of macrophages were incubated with **(A)** aniline blue to evaluate the contents of glucans; **(C)** ConA Type VI conjugated to FITC to evaluate glycosylated proteins and **(E)** CFW to evaluate chitin amount in the cell wall. **(B, D, F)** Fluorescence intensity graph. The values of fluorescence intensity (in pixels) and the standard error of each analysis were used to plot the graph. Data are expressed as mean ± standard error (represented using error bars). ^∗^Significantly different comparison between treated cells in a *P*-value of ≤0.05. All representative images were magnified 400×.

Proteomic data were confirmed by fluorescence microscopy using aniline blue, ConA, and CFW as markers for glucans, carbohydrates residues in proteins (predominantly), and chitin, respectively (Figure 5). *P. brasiliensis* yeast cells recovered from non-primed macrophages presented higher fluorescence intensity of aniline blue, ConA, and CFW, suggesting higher content of glucans, carbohydrates residues from proteins, and chitin compared to fungal cells recovered from primed macrophages (Figure 5).

### *P. brasiliensis*, *Pb*18 Shows Higher Mitochondrial Activity in Non-primed Macrophage Infection

**FIGURE 6:**
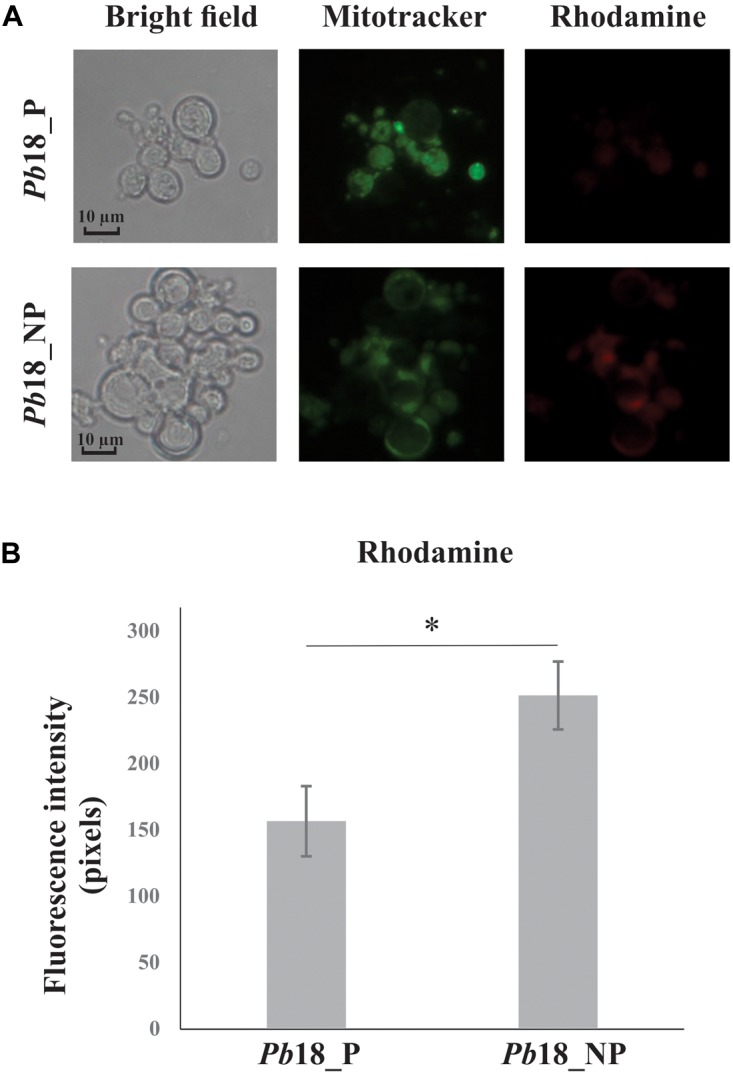
Mitochondrial activity of *P. brasiliensis*, *Pb*18 recovered from alveolar macrophages. **(A)** *Pb*18 yeast cells recovered from primed (*Pb*18_P) and non-primed macrophages (*Pb*18_NP) were incubated with MitoTracker Green FM and rosamine Mitotracker probes. The mitochondrial activity was evaluated by using rhodamine as a dye for mitochondrial membrane potential. **(B)** The rhodamine fluorescence intensity was measured using the AxioVision Software (Carl Zeiss). The values of fluorescence intensity (in pixels) and the standard deviation of each analysis were used to plot the graph. Data are expressed as mean ± standard deviation (represented using error bars) of the minimum of 100 cells for each microscope slide, in triplicates, for each condition. ^∗^Significantly different comparison between treated cells in a *P*-value of ≤0.05. All representative images were taken using an Axioscope (Carl Zeiss) microscope and 400× magnified.

Increase of proteins related to the electron transport chain and ATP synthesis complex were observed in yeast cells recovered from primed and non-primed macrophages. We used MitoTracker Green FM as a total mitochondrial dye (green) and the mitochondrial activity was evaluated by using rhodamine dye (red), which stains selectively according to mitochondrial membrane potential, as depicted in Figure 6. The fluorescence intensity of rhodamine was significantly increased in *Pb*18_NP compared to *Pb*18_P.

## Discussion

Despite the similarity in the adaptation pattern of *Pb*18 cells in primed and non-primed macrophages at 6 h post infection, our results could suggest that metabolic ability to regulate pathways and proteins important to ensure fungal survival is diminished in the fungus after interaction with primed macrophages. Many adaptation strategies have been described as essential for the viability of fungi under nutrient deprivation conditions, such as up regulation of gluconeogenesis, fatty acids degradation, glyoxylate cycle (Seider et al., 2014).

The intracellular environment imposes numerous difficulties to pathogens, such as the action of hydrolytic enzymes, nutrient deprivation, and presence of reactive species of oxygen and nitrogen (Haass et al., 2007). To establish the infection, pathogens require a metabolic flexibility to assimilate the available nutrients and a powerful antioxidant system that allows them to survive intracellularly (Seider et al., 2014; Kasper et al., 2015; Camacho and Nino-Vega, 2017). The cells of the innate immune system, mainly macrophages and neutrophils, are the primary line of defense against *Paracoccidioides* infection (Franco, 1987; Calich et al., 2008; Mendes-Giannini et al., 2008). However, microscopic findings show that *Paracoccidioides* is able to survive and multiply inside phagocytes, although this ability is inhibited by the activation of these cells with IFN-γ (Brummer et al., 1988; Rodrigues et al., 2007). Those findings were confirmed in our work, where we determined a time-course survival of *P. brasiliensis* during interaction with non-primed macrophages. The cells primed with INF-γ showed increase in the adherence/internalization index in the first 6 h of interaction, while the adherence/internalization of the fungus in non-primed macrophages was affected only after 9 h of infection (Figure 1A). This difference in the fungicidal potential between primed and non-primed phagocytes was observed in infection assays with several *Paracoccidioides* isolates. The addition of INF-γ did not increase the phagocytosis index, but it confers microbicidal activity to the macrophages in a dose-dependent manner, as described (Brummer et al., 1988, 1989).

Previous proteomic and transcriptomic studies revealed that pathogenic bacteria and fungi reprogram their metabolism, regulating negatively the glycolytic pathway and activating alternative routes of carbon consumption, as gluconeogenesis, amino acid degradation, fatty acids oxidation, glyoxylate cycle, and ethanol production, during macrophage infection (Sprenger et al., 2017). Our analysis revealed that *P. brasiliensis* was able to modify its metabolism in both conditions, intracellular environment of primed and non-primed macrophages with INF-γ. However, *P. brasiliensis* stress response is most evident during interaction with the non-primed macrophages with IFN-γ, suggesting fungal cells ability to respond and survive during interaction with non-primed macrophages is increased compared to primed-macrophages (Supplementary Tables 2, 3). The fungus adaptation during interaction with non-primed macrophages is also more evident when we analyzed the pentose-phosphate pathway, methylcitrate cycle, and synthesis of cell wall precursors (Figure 3, 4; Supplementary Table 3). The activation of those pathways in *P. brasiliensis* after interaction with non-primed macrophages may favor the fungus survival in the hostile environment in the macrophages. The pentose-phosphate pathway also contributes to the defense against oxidative stress since it provides NADPH. The methylcitrate synthase is essential for the degradation of toxic compounds as propionyl-CoA, and is necessary for the pathogen *A. fumigattus* to establish infection in murines (Ibrahim et al., 2008). The up-regulation of those pathways was also observed in *P. brasiliensis* during 6 h of murine lung infection and after 24 h of infection in J774 macrophages (Parente-Rocha et al., 2015; Pigosso et al., 2017). Those metabolic peculiarities indicate that the intracellular environment of primed macrophages prevents the fungus from rapidly activating strategic pathways of metabolic adaptation, as it does in non-primed macrophages.

It was possible to observe the accumulation of glycolytic enzymes that can generate precursors of *Paracoccidioides* cell wall components, in *P. brasilensis* recovered of non-primed macrophages (Table 2). Proteomic data were confirmed by fluorescence microscopy, since higher amounts of glucans, glycosylated proteins, and chitin were observed in the cell wall of yeasts recovered from non-primed macrophages (Figure 5).

Our data corroborated studies previously conducted (Brummer et al., 1989) in which the morphology of *P. brasiliensis* presented differences after internalization by primed and non-primed peritoneal macrophages. During infection of primed macrophages, it was observed deterioration of the fungus cell wall until complete digestion and elimination, while in non-primed macrophages the *P. brasiliensis* cell wall remained intact. *C. neoformans* also depicted induction in the expression of genes encoding enzymes involved in polysaccharide synthesis, during infection in primed macrophages infection, which may be associated with the formation of cell wall or capsule components (Fan et al., 2005). Our results suggest that the cell wall maintenance and remodeling probably occurs because non-primed macrophage allows the survival and multiplication of the fungus.

A higher abundance of proteins related to electron carrier chain and ATP synthesis was detected in *P. brasiliensis* derived from non-primed macrophages (Figure 2 and Table 3). We evaluated the mitochondrial activity of *P. brasiliensis* by labeling yeast cells with mitotracker and rhodamine. The latter, dyes mitochondria according to membrane potential. The fluorescence intensity of rhodamine was higher in yeast cells during infection of non-primed macrophages, which indicates an increased mitochondrial activity (Figure 6). Among the functions performed by mitochondria, we can highlight the supply of cellular energy, cross-talk between pro-survival and pro-death pathways, and also role in the response to metabolic stress (Nunnari and Suomalainen, 2012). A study with *Aspergillus nidulans* demonstrated that the mutant strain of a gene related to mitochondrial function and cellular respiration presented a decrease in the mass and function of mitochondria and of oxidative phosphorylation process, which influenced glucose uptake. This deletion also led to an increase in endogenous ROS levels, which is toxic to the cell (Krohn et al., 2014).

**Table 3:** Up-regulated proteins related with mitochondrial activity in *Pb*18_A and *Pb*18_NA.

Accession number^1^	Protein description	*Pb*18_P^2^	*Pb*18_NP^2^
**ATP synthesis**			
PADG_04729	ATP synthase subunit D, mitochondrial	2.09	3.24
PADG_07042	ATP synthase F1, delta subunit	^∗^	*^∗^*
PADG_07813	ATP synthase F1, gamma subunit	2.38	2.02
PADG_08349	ATP synthase subunit beta, mitochondrial	3.38	2.92
PADG_07789	ATP synthase subunit delta, mitochondrial	1.84	2.53
PADG_07964	Vacuolar ATP synthase subunit E	ND	1.52
**Electron transport and membrane-associated energy conservation**			
PADG_11981	V-type proton ATPase catalytic subunit A	2.36	1.81
PADG_04319	V-type ATPase, G subunit	1.75	2.08
PADG_00688	F-type H+-transporting ATPase subunit H	2.11	2.56
PADG_03175	V-type proton ATPase subunit F	1.87	2.88
PADG_08391	Plasma membrane ATPase	ND	1.41
PADG_07081	Electron transfer flavoprotein subunit alpha	ND	1.74
PADG_11468	Electron transfer flavoprotein beta-subunit	^∗^	*^∗^*
PADG_06978	Cytochrome C	ND	1.68
PADG_04397	Cytochrome c oxidase subunit 4, mitochondrial	2.88	3.55
PADG_05750	Putative cytochrome c oxidase subunit Via	2.5	3.89
PADG_02745	NADH-ubiquinone oxidoreductase Fe–S protein 6	ND	*^∗^*
PADG_07749	NAD(P)H:quinone oxidoreductase, type IV	1.59	2.34
PADG_01366	NADH-ubiquinone oxidoreductase 1 alpha subcomplex subunit 5	1.82	2.21

*^1^Accession number obtained in the Paracoccidioides database available at http://www.broadinstitute.org/annotation/genome/paracoccidioides_brasiliensis/MultiHome.html. ^2^Ratio values were obtained by dividing the values of protein abundance (in fmol) from Pb18 during infection of activated and non-activated macrophages, by the abundance in control. Proteins with a minimum fold change of 40% were considered regulated. ^∗^Proteins detected in P. brasiliensis Pb18 only during respective macrophage infection. ND, non-detected proteins.*

Interestingly, gluconeogenesis has been described as a crucial strategy during the first steps of *C. neoformans* infection, whereas glycolysis has importance later, being fundamental for the permanence of the pathogen in the host (Price et al., 2011). Our proteomic analyzes showed that, in primed and non-primed macrophage infection, *Pb*18 cells did not exhibit up-regulation of gluconeogenesis (Supplementary Tables 2, 3). This data are also observed in *P. brasiliensis* after 6 h of lung murine infection; the authors suggest the occurrence of glucose reserve, since the fungus was grown in a nutrient rich medium prior to infection (Pigosso et al., 2017). In this sense, activation of gluconeogenesis has been observed in *Pb*18 after a longer period of infection in macrophages (Parente-Rocha et al., 2015).

The fungus also increased the expression of proteins involved in cell rescue, defense, and virulence in both conditions (Table 1). Autophagic proteins as serine proteinase (PADG_07422), vacuolar aminopeptidase (PADG_07460), carboxypeptidase Y (PADG_06314) were up-regulated. These enzymes have been described as important virulence factors that promote the recycling of cytoplasmic components in pathogen starvation mode. Autophagic processes have been described in *S. cerevisiae* and *Candida glabrata* as a way of survival in the host and during nitrogen starvation and depletion of nutrients (Inoue and Klionsky, 2010; Roetzer et al., 2010). Serine proteinase has recently been described in *P. brasiliensis* as a virulence factor that favors survival upon nitrogen deprivation, as well as tissue invasion, since it is secreted in large amounts in the host during lung murine infection (Parente et al., 2010; Pigosso et al., 2017).

Here, it was also observed, an increased expression of heat shock proteins and proteins involved in detoxification and stress response, as TrxR (PADG_01551) and SOD (PADG_01954 and PADG_07418) in *P. brasiliensis* recovered of primed and non-primed macrophages, although the higher expression was observed in the last condition (Table 1). TrxR, which works in conjunction with thioredoxin, is induced in *Paracoccidioides* during oxidative stress and infection assays (Table 1). The TrxR, which works in conjunction with thioredoxin, is induced in *Paracoccidioides* during oxidative stress and infection assays (Grossklaus et al., 2013; Pigosso et al., 2017). TrxR is a molecule of antioxidant resistance, since it binds to NADPH and reduces thioredoxin, forming thioredoxin disulfide and NADP^+^; the final reaction contributes to the removal of superoxide radicals (Yoshida et al., 2003; Missall and Lodge, 2005). Some fungi such as members of *Candida* species increase the expression of the thioredoxin system during oxidative and nitrosative stresses (Brown et al., 2009). In *C. neoformans*, the action of this enzyme also gives high virulence to the fungus. This enzyme has called attention as a possible target of antifungal drugs against *Paracoccidioides* infection (Missall and Lodge, 2005; Abadio et al., 2015).

In synthesis, all the data indicate that the *P. brasiliensis* adapts rapidly within not primed macrophages, where it can multiply and probably spread through the tissues of the host. The data corroborate with clinical data, that report patients with acute or chronic PCM presenting low production of phagocyte activating cytokines, such as INF-γ, IL-12, and TNF-α (Benard et al., 2001). These cytokines play an important role in the microbicidal activity of macrophages, recruitment of defense cells to the site of infection and formation of efficient granulomas to contain the dissemination of *Paracoccidioides* (Nishikaku et al., 2011). This comparative proteomic study contributes to the understanding of factors that can lead to the inhibition or evolution of PCM as well as its pathogenesis.

## Concluding Remarks

Comparative proteomic analysis of *P. brasiliensis* during phagocyte infection revealed metabolic peculiarities that favor the survival of *P. brasiliensis* in the intracellular environment of non-primed macrophages. In both conditions, primed and non-primed macrophages, the fungus increased the expression of enzymes related to amino acid degradation, TCA and glyoxylate cycles, antioxidant enzymes, and virulence factors. However, activation of the pentose-phosphate pathway, methylcytrate cycle, synthesis of cell wall precursors, and intense mitochondrial activity was observed mainly in yeast cells recovered from non-primed macrophages. These pathways may favor the viability of the fungus compared to yeasts internalized by primed phagocytes. Considering the obtained data, we could suggest that, compared to primed macrophages, non-primed macrophages allow a more permissive environment to *P. brasiliensis* to adapt to the host milieu.

## Author Contributions

CS conceived and finalized the manuscript. EC and DA performed the experiments. LB carried out proteomic data. JP-R contributed in the experiments design and performed the heat maps. EC, DA, LB, JP-R, CB, MO, and CS designed the study, discussed, analyzed, and interpreted the data and wrote the manuscript.

## Conflict of Interest Statement

The authors declare that the research was conducted in the absence of any commercial or financial relationships that could be construed as a potential conflict of interest.

## Footnotes

1 http://www.uniprot.org

2 http://mips.helmholtz-muenchen.de/funcatDB/

3 https://www.ncbi.nlm.nih.gov/

4 http://en.bio-soft.net/chip/MeV.html
