# Supplementary figures and images for: Proteomic Analysis of Paracoccidioides brasiliensis During Infection of Alveolar Macrophages Primed or Not by Interferon-Gamma
Source: Front Microbiol. 2019 Feb 5;10:96. doi: 10.3389/fmicb.2019.00096 (PMC6371752; doi:10.3389/fmicb.2019.00096)

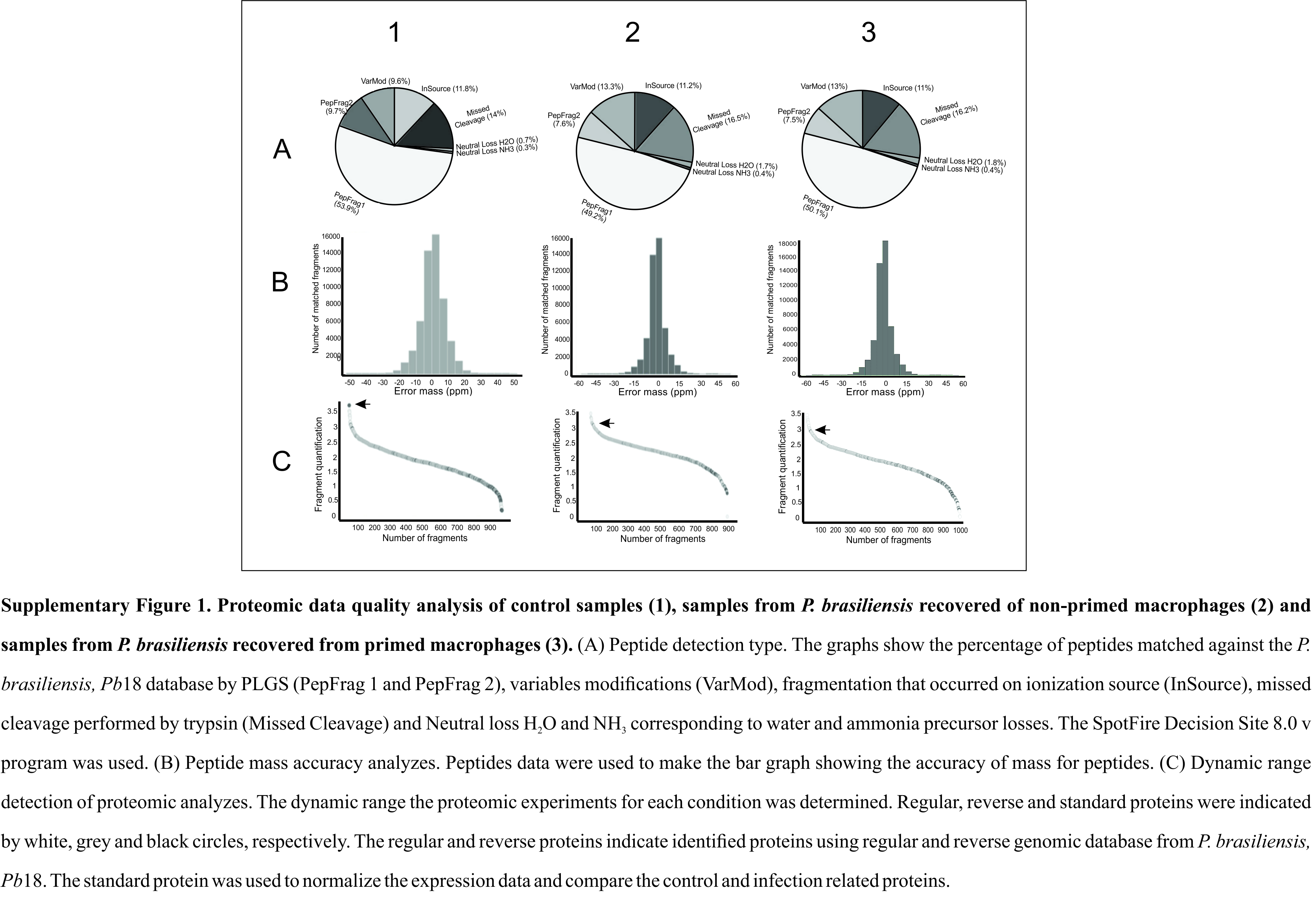

Supplement: Supplementary file 1 [file Image_1.JPEG]

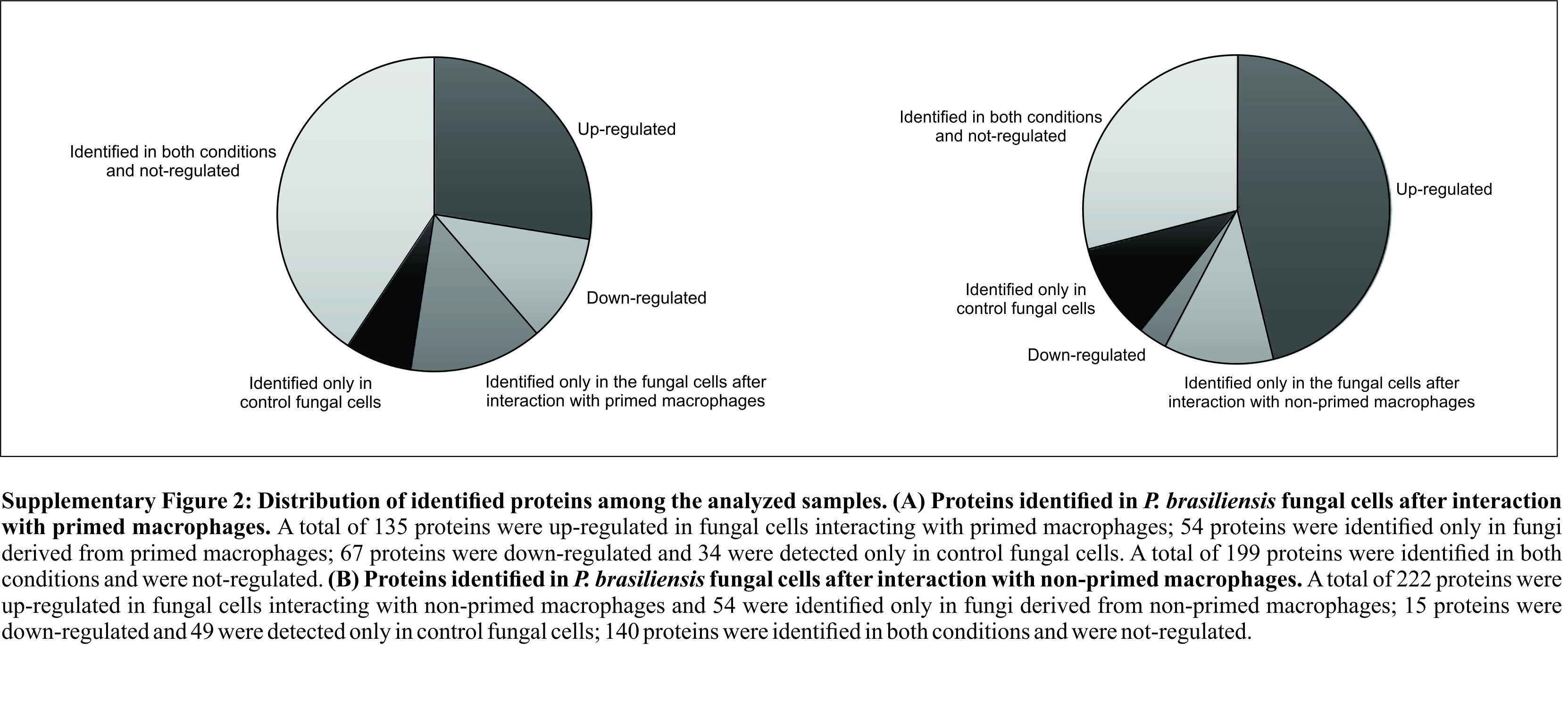

Supplement: Supplementary file 2 [file Image_2.JPEG]
