# Supplementary material for: Proteomic Analysis of Paracoccidioides brasiliensis During Infection of Alveolar Macrophages Primed or Not by Interferon-Gamma
Source: Front Microbiol. 2019 Feb 5;10:96. doi: 10.3389/fmicb.2019.00096 (PMC6371752; doi:10.3389/fmicb.2019.00096)
Supplement: Supplementary file 4 [file Table_2.docx]

**Supplementary Table 2. Regulated proteins of *P. brasiliensis*, *Pb*18 after 6 h infection, in INF-γ activated alveolar macrophages**

| **Accession number^1^** | **Protein description** | **Score^2^** | ***Pb*18_P x CTRL**  **Ratio^3^** | |
| --- | --- | --- | --- | --- |
| **Functional categories^4^** | |  |  | |
| **AMINO ACID METABOLISM** | |  |  | |
| **Amino acid degradation** | |  |  | |
| PADG_00822 | glutaminase A | 651.49 | *** | |
| PADG_04686 | glutamine synthetase | 496.27 | *** | |
| PADG_05922 | glutamate carboxypeptidase | 3500.33 | 1.75 | |
| PADG_04516 | NADP-specific glutamate dehydrogenase | 651.57 | 1.59 | |
| PADG_05085 | 1-pyrroline-5-carboxylate dehydrogenase | 3215.32 | 1.44 | |
| PADG_06546 | puromycin-sensitive aminopeptidase | 499.08 | 1.59 | |
| PADG_04689 | N-acetyl-gamma-glutamyl-phosphate reductase | 686.12 | *** | |
| PADG_02214 | 4-aminobutyrate aminotransferase | 4656.87 | 0.44 | |
| PADG_03627 | 2-oxoisovalerate dehydrogenase subunit beta | 674.79 | 0.64 | |
| PADG_03466 | 3-hydroxyisobutyrate dehydrogenase | 504.5 | + | |
| PADG_07369 | isovaleryl-CoA dehydrogenase | 3828.73 | 0.52 | |
| PADG_07370 | methylcrotonoyl-CoA carboxylase beta chain | 1047.22 | 0.6 | |
| PADG_00637 | arginase | 1161.3 | 0.37 | |
| PADG_01418 | cysteine dioxygenase | 767.22 | *+* | |
| PADG_03686 | aspartate aminotransferase | 395.76 | + | |
| PADG_00832 | adenylosuccinate synthetase | 969.84 | + | |
| PADG_01718 | saccharopine dehydrogenase [NADP+, L-glutamate-forming] | 584.09 | + | |
| PADG_08468 | 4-hydroxyphenylpyruvate dioxygenase | 5519.57 | 0.71 | |
| PADG_08464 | maleylacetoacetate isomerase | 1624.24 | 0.71 | |
| PADG_00215 | aromatic-L-amino-acid decarboxylase | 579.63 | + | |
| PADG_08466 | homogentisate 1,2-dioxygenase | 1254.23 | 0.53 | |
| **Amino acid biosynthesis** | |  |  | |
| PADG_07029 | acetylornithine aminotransferase | 522.48 | *** | |
| PADG_00888 | argininosuccinate synthase | 652.7 | 1.97 | |
| PADG_01615 | homocitrate synthase, mitochondrial | 559.95 | 3.18 | |
| PADG_08376 | aspartate-semialdehyde dehydrogenase | 696.7 | 1.68 | |
| PADG_05111 | serine hydroxymethyltransferase, cytosolic | 1716.94 | 2.01 | |
| PADG_02914 | glycine cleavage system T protein | 1568.09 | 1.4 |  |
| PADG_05896 | phosphoglycerate dehydrogenase | 313.7 | *** | |
| PADG_02726 | cysteine synthase | 898.45 | 1.43 | |
| PADG_03522 | methylthioadenosine phosphorylase | 479.79 | 1.44 | |
| PADG_08662 | cystathionine beta-lyase | 398.91 | *** | |
| PADG_08328 | 5-methyltetrahydropteroyltriglutamate-homocysteine S-methyltransferase | 4196.01 | 1.89 | |
| PADG_07907 | acetolactate synthase | 1231.54 | 1.46 | |
| PADG_07609 | dihydroxy-acid dehydratase | 505.55 | 2.18 | |
| PADG_03114 | 3-deoxy-7-phosphoheptulonate synthase | 523.88 | *** | |
| **NITROGEN METABOLISM** | |  |  | |
| PADG_06490 | formamidase | 1170.68 | 1.47 | |
| PADG_07010 | urease accessory protein UreG | 643.89 | *** | |
| **NUCLEOTIDE/NUCLEOSIDE/NUCLEOBASE METABOLISM** | |  |  | |
| PADG_08066 | purine nucleoside phosphorylase I, inosine and guanosine-specific | 1433.7 | 1.46 | |
| PADG_01424 | inosine-uridine preferring nucleoside hydrolase | 435.72 | *** | |
| PADG_02246 | adenosine kinase | 1864.44 | 1.43 | |
| PADG_04828 | adenylosuccinate lyase | 614.73 | 1.91 | |
| PADG_06297 | phosphoribosylamine-glycine ligase | 410.55 | *** | |
| PADG_07585 | inosine-5'-monophosphate dehydrogenase | 914.88 | 1.8 | |
| PADG_07970 | dihydroorotase, homodimeric type | 580.91 | *** | |
| PADG_04288 | endoribonuclease L-PSP | 22381.84 | 0.67 | |
| **C-COMPOUND AND CARBOHYDRATE METABOLISM** | |  |  | |
| PADG_04374 | UTP-glucose-1-phosphate uridylyltransferase | 722.26 | *** | |
| PADG_11132 | Phosphoglucomutase | 367.02 | *** | |
| PADG_03943 | Phosphomannomutase | 388.32 | *** | |
| PADG_04761 | mannosyl-oligosaccharide glucosidase | 667.61 | 1.84 | |
| PADG_07523 | neutral alpha-glucosidase AB | 600.23 | 1.91 | |
| PADG_06221 | formate dehydrogenase | 632.87 | + | |
| **PENTOSE-PHOSPHATE PATHWAY** | |  |  | |
| PADG_03651 | 6-phosphogluconate dehydrogenase, decarboxylating 1 | 567.53 | 2.27 | |
| PADG_07420 | transaldolase | 3551.69 | 0.71 | |
| **GLYCOLYSIS/GLUCONEOGENESIS** | |  |  | |
| PADG_03813 | Hexokinase | 587.08 | 2.45 | |
| PADG_07950 | glucokinase | 433.94 | 1.78 | |
| PADG_00451 | glucose-6-phosphate isomerase | 919.44 | 1.87 | |
| PADG_05109 | 2,3-bisphosphoglycerate-independent phosphoglycerate mutase | 1255.36 | 1.71 | |
| PADG_08503 | phosphoenolpyruvate carboxykinase | 3649.92 | 1.55 | |
| PADG_02411 | glyceraldehyde-3-phosphate dehydrogenase | 29527.63 | 0.42 | |
| PADG_01896 | phosphoglycerate kinase | 6553.54 | 0.58 | |
| **PYRUVATE METABOLISM** | |  |  | |
| PADG_01797 | pyruvate dehydrogenase protein X component | 576.86 | 2.05 | |
| PADG_04165 | pyruvate dehydrogenase complex component Pdx1 | 691.24 | 1.43 | |
| PADG_06494 | dihydrolipoyl dehydrogenase | 3577,44 | 1.4 | |
| PADG_00714 | pyruvate decarboxylase | 2477.21 | 0.61 | |
| PADG_03276 | S-(hydroxymethyl)glutathione dehydrogenase | 1082.77 | + | |
| **FERMENTATION** |  |  |  | |
| PADG_00171 | L-lactate dehydrogenase | 3625.04 | 0.71 | |
| **TRICARBOXYLIC ACID CYCLE** | |  |  | |
| PADG_08387 | citrate synthase, mitochondrial | 665.29 | 1.84 | |
| PADG_04994 | citrate synthase subunit 1 | 2691.59 | 0.63 | |
| PADG_11845 | aconitate hydratase, mitochondrial | 2588.05 | 1.76 | |
| PADG_00317 | succinyl-CoA ligase subunit beta | 356.85 | 4.09 | |
| PADG_01762 | oxoglutarate dehydrogenase (succinyl-transferring), E1 component | 566.44 | 1.40 | |
| PADG_00052 | succinate dehydrogenase [ubiquinone] flavoprotein subunit, mitochondrial | 1339.48 | 1.61 | |
| PADG_08013 | succinate dehydrogenase [ubiquinone] iron-sulfur subunit, mitochondrial | 768.68 | 2.48 | |
| PADG_08119 | fumarate hydratase, mitochondrial | 596.3 | 2.31 | |
| **METHYLCITRATE CYCLE** | |  |  | |
| PADG_04718 | 2-methylcitrate dehydratase | 11210.66 | 1.55 | |
| PADG_04709 | methyl-isocitrate lyase | 900.43 | 1.55 | |
| PADG_04710 | 2-methylcitrate synthase, mitochondrial | 1868.25 | 0.65 | |
| **GLYOXALATE CYCLE** | |  |  | |
| PADG_01483 | isocitrate lyase | 1754.4 | 1.4 | |
| **ELECTRON TRANSPORT AND RESPIRATION** | |  |  | |
| PADG_01366 | NADH-ubiquinone oxidoreductase | 3175.48 | 1.82 | |
| PADG_07749 | NAD(P)H:quinone oxidoreductase, type IV | 41840.44 | 1.59 | |
| PADG_05750 | putative cytochrome c oxidase subunit Via | 4381.04 | 2.5 | |
| PADG_04397 | cytochrome c oxidase subunit 4, mitochondrial | 1429.9 | 2.88 | |
| PADG_11468 | electron transfer flavoprotein beta-subunit | 471.59 | *** | |
| PADG_11981 | V-type proton ATPase catalytic subunit A | 430.31 | 2.36 | |
| PADG_03175 | V-type proton ATPase subunit F | 877.44 | 1.87 | |
| PADG_04319 | V-type ATPase, G subunit | 448.84 | 1.75 | |
| PADG_00688 | F-type H+-transporting ATPase subunit H | 2589.81 | 2.11 | |
| PADG_08391 | plasma membrane ATPase | 442.15 | 1.76 | |
| PADG_08394 | cytochrome b-c1 complex subunit 2 | 904.65 | 0.61 | |
| **ATP SYNTHESIS** | |  |  | |
| PADG_07042 | ATP synthase F1, delta subunit | 514.91 | *** | |
| PADG_04729 | ATP synthase subunit D, mitochondrial | 2219.02 | 2.09 | |
| PADG_07813 | ATP synthase F1, gamma subunit | 1367.33 | 2.38 | |
| PADG_08349 | ATP synthase subunit beta, mitochondrial | 17882.4 | 3.38 | |
| PADG_07789 | ATP synthase subunit delta, mitochondrial | 2026.9 | 1.84 | |
| **FATTY ACID BIOSYNTHESIS** | |  |  | |
| PADG_00255 | fatty acid synthase subunit beta dehydratase | 889.54 | 1.75 | |
| PADG_05783 | farnesyl pyrophosphate synthetase | 3596.45 | *** | |
| **DEGRADATION OF KETONE BODIES** | |  |  | |
| PADG_04939 | succinyl-CoA:3-ketoacid-coenzyme A transferase subunit B | 1703.1 | 1.68 | |
| **OXIDATION OF FATTY ACIDS** | |  |  | |
| PADG_01209 | enoyl-CoA hydratase | 5708.9 | 0.36 | |
| PADG_03194 | 3-ketoacyl-CoA thiolase B | 2030.96 | 0.57 | |
| PADG_01687 | 3-ketoacyl-CoA thiolase | 3955.45 | 0.67 | |
| PADG_03449 | isopentenyl-diphosphate delta-isomerase | 795.96 | + | |
| PADG_04343 | short chain dehydrogenase/reductase | 620.43 | + | |
| PADG_02751 | acetyl-CoA acetyltransferase | 2879.73 | 0.41 | |
| **METABOLISM OF VITAMINS. COFACTORS. AND PROSTHETIC GROUPS** | |  |  | |
| PADG_00443 | dihydropteroate synthase | 1863.88 | 1.52 | |
| PADG_01886 | adenosylhomocysteinase | 3209.32 | 1.69 | |
| PADG_00513 | 2-succinylbenzoate-CoA ligase | 567.59 | *** | |
| **SECONDARY METABOLISM** | |  |  | |
| PADG_04899 | metallo-beta-lactamase domain-containing protein | 1103.5 | *** | |
| PADG_08108 | coproporphyrinogen III oxidase | 526.04 | *** | |
| PADG_04636 | dienelactone hydrolase family protein | 1042.89 | 1.43 | |
| PADG_04175 | inorganic pyrophosphatase | 2372.74 | 1.41 | |
| PADG_02981 | ThiJ/PfpI family protein | 2110.74 | 0.65 | |
| **SIGNAL TRANSDUCTION** | |  |  | |
| PADG_02017 | calmodulin | 917.7 | 2.58 | |
| PADG_01565 | calnexin | 1309.53 | 1.89 | |
| PADG_01243 | rab GDP-dissociation inhibitor | 838.81 | 2.15 | |
| PADG_05517 | rho GDP-dissociation inhibitor | 620.8 | 2.69 | |
| PADG_04440 | 14-3-3-like protein 2 | 22781.86 | 1.41 | |
| PADG_05608 | GTP-binding protein ypt7 | 512.66 | *** | |
| PADG_03544 | ser/Thr protein phosphatase family protein | 456.22 | 1.99 | |
| PADG_11275 | CMGC/MAPK protein kinase | 439.81 | + | |
| PADG_00172 | ras-like GTP-binding protein | 506.63 | + | |
| **CYTOSKELETON** | |  |  | |
| PADG_00945 | actin like protein 2/3 complex, subunit 5 | 1210.25 | 1.52 | |
| PADG_00422 | actin cytoskeleton protein (VIP1) | 3501.22 | 1.69 | |
| PADG_05538 | actin | 517.74 | 1.99 | |
| PADG_05239 | tubulin-specific chaperone Rbl2 | 949.14 | 1.64 | |
| **CELL CYCLE** | |  |  | |
| PADG_05683 | cell division control protein 48 | 3099.11 | 1.95 | |
| PADG_05875 | centromere/microtubule-binding protein cbf5 | 473.9 | *** | |
| PADG_04056 | 14-3-3 family protein épsilon | 11315.76 | 1.43 | |
| PADG_07930 | ARP2/3 complex 20 kDa subunit | 1565.37 | *** | |
| PADG_07733 | ARP2/3 complex 34 kDa subunit | 693.88 | + | |
| PADG_02763 | cyclin-dependent kinase regulatory subunit | 763.95 | + | |
| **DNA PROCESSING** | |  |  | |
| PADG_02683 | UV excision repair protein Rad23 | 1951.91 | 1.58 | |
| PADG_00656 | non-histone chromosomal protein 6 | 6855.17 | 1.64 | |
| PADG_00718 | histone chaperone asf1 | 625.3 | 1.89 | |
| PADG_00872 | Histone | 3651.58 | 3.45 | |
| PADG_00873 | histone H3 | 2817.56 | 3.93 | |
| PADG_07134 | histone H4 | 3673.03 | 4.13 | |
| PADG_08423 | RuvB-like helicase 2 | 443.26 | *** | |
| **TRANSCRIPTION** | |  |  | |
| PADG_11711 | ATP-dependent RNA helicase eIF4A | 523.13 | 3.59 | |
| PADG_00067 | polymerase II polypeptide D | 1667.1 | *** | |
| PADG_01151 | DNA-directed RNA polymerase II subunit RPB11 | 475.45 | *** | |
| PADG_04657 | nascent polypeptide-associated complex subunit beta | 2060.21 | 3.32 | |
| PADG_04730 | nascent polypeptide-associated complex subunit alpha | 2511.44 | 1.89 | |
| PADG_07888 | eukaryotic translation initiation factor 5A | 938.57 | *** | |
| PADG_02825 | small nuclear ribonucleoprotein Lsm8 | 936.12 | 1.52 | |
| PADG_03696 | nuclear polyadenylated RNA-binding protein Nab2 | 580.16 | *** | |
| PADG_04981 | nucleoporin p58/p45 | 881.57 | *** | |
| PADG_05393 | mRNA decapping hydrolase | 487.75 | 2.8 | |
| PADG_04672 | ATP-dependent RNA helicase SUB2 | 1324.65 | *** | |
| PADG_04796 | pre-mRNA-splicing factor rse1 | 687.99 | *** | |
| PADG_06734 | U6 snRNA-associated Sm-like protein LSm7 | 2200.35 | *** | |
| PADG_00676 | RNApii degradation factor def1 | 568.85 | 1.89 | |
| PADG_11958 | small nuclear ribonucleoprotein | 860.29 | *** | |
| PADG_11062 | bZIP transcription factor | 817.23 | 2.22 | |
| **PROTEIN SYNTHESIS** | |  |  | |
| PADG_02759 | ribosome recycling factor | 787.37 | 3.42 | |
| PADG_00784 | 40S ribosomal protein S0 | 4550.38 | 1.43 | |
| PADG_00995 | ubiquitin-40S ribosomal protein S27a | 2933.72 | 2.33 | |
| PADG_02056 | ribosomal protein L7/L12 | 6872.26 | 1.93 | |
| PADG_02446 | 60S acidic ribosomal protein P2 | 14965.78 | 5.00 | |
| PADG_04030 | 60S acidic ribosomal protein P0 | 1428.5 | 1.89 | |
| PADG_05939 | 60S ribosomal protein L27a | 1823.21 | 1.8 | |
| PADG_07891 | ubiquitin-60S ribosomal protein L40 | 2561.7 | 2.58 | |
| PADG_08244 | 60S acidic ribosomal protein P1 | 14587.27 | 1.58 | |
| PADG_08605 | 40S ribosomal protein S28 | 5195.73 | 1.71 | |
| PADG_02206 | DnaJ domain protein Psi | 707.66 | 1.5 | |
| PADG_01949 | translation elongation factor Tu | 3152.3 | 1.64 | |
| PADG_02296 | eukaryotic translation initiation factor 3 subunit F | 1272.06 | *** | |
| PADG_04083 | eukaryotic translation initiation factor 2 subunit gamma | 904.31 | *** | |
| PADG_07977 | eukaryotic translation initiation factor 1A, Y-chromosomal | 703.74 | *** | |
| PADG_08033 | eukaryotic translation initiation factor 3 subunit B | 557.3 | 1.63 | |
| PADG_01558 | histidyl-tRNA synthetase | 469.31 | 2.03 | |
| PADG_07582 | phenylalanine-tRNA ligase, alpha subunit | 691.71 | 1.75 | |
| PADG_00066 | tRNA (guanine(37)-N1)-methyltransferase | 384.07 | *** | |
| PADG_00333 | 40S ribosomal protein S16 | 1144.09 | 0.71 | |
| PADG_00335 | 40S ribosomal protein S14 | 4602.27 | 0.66 | |
| PADG_00354 | 40S ribosomal protein S17 | 1524.81 | 0.51 | |
| PADG_00942 | 40S ribosomal protein S7 | 4390.69 | 0.63 | |
| PADG_01083 | 60S ribosomal protein L32 | 1109.4 | 0.44 | |
| PADG_01267 | 40S ribosomal protein S11 | 5706.97 | 0.41 | |
| PADG_01281 | mitochondrial 37S ribosomal protein MRPS8 | 463.22 | + | |
| PADG_01407 | 40S ribosomal protein | 7497.56 | 0.62 | |
| PADG_01654 | 40S ribosomal protein S6-A | 2963.94 | 0.58 | |
| PADG_01914 | 60S ribosomal protein L35 | 1408.15 | 0.67 | |
| PADG_02445 | 40S ribosomal protein S15 | 6405.8 | 0.58 | |
| PADG_02797 | mitochondrial 54S ribosomal protein YmL3 | 428.68 | + | |
| PADG_03315 | 40S ribosomal protein S4 | 3899.5 | 0.58 | |
| PADG_03326 | 40S ribosomal protein S9 | 4494.77 | 0.39 | |
| PADG_03778 | 60S ribosomal protein L10-A | 3782.16 | 0.55 | |
| PADG_03781 | 60S ribosomal protein L30 | 2485.54 | 0.71 | |
| PADG_12253 | 60S ribosomal protein L3 | 1750.4 | 0.63 | |
| PADG_12365 | 40S ribosomal protein S8-A | 2524.96 | 0.53 | |
| PADG_03856 | 60S ribosomal protein L15 | 2106.51 | 0.48 | |
| PADG_04106 | 60S ribosomal protein L11 | 4743.63 | 0.50 | |
| PADG_07469 | RNase III domain-containing protein | 465.89 | + | |
| PADG_04848 | 60S ribosomal protein L8-B | 6589.56 | 0.68 | |
| PADG_05025 | ribosomal protein L24 | 1716.08 | + | |
| PADG_05338 | 60S ribosomal protein L18-B | 4386.56 | 0.2 | |
| PADG_06313 | 40S ribosomal protein S18 | 2306.16 | 0.57 | |
| PADG_06525 | 40S ribosomal protein S1 | 4906.3 | 0.64 | |
| PADG_06680 | 40S ribosomal protein S2 | 3503.9 | 0.61 | |
| PADG_06838 | 40S ribosomal protein S5 | 5075.18 | 0.53 | |
| PADG_07583 | 40S ribosomal protein S21 | 3306.23 | 0.65 | |
| PADG_07803 | 60S ribosomal protein L12 | 4692.42 | 0.65 | |
| PADG_07870 | 30S ribosomal protein S7 | 638.76 | + | |
| PADG_08213 | ribosomal protein S2 | 593.74 | + | |
| PADG_08602 | 40S ribosomal protein S2 | 3621.3 | 0.46 | |
| PADG_11832 | 60S ribosomal protein L31 | 3256.39 | 0.39 | |
| PADG_00355 | 40S ribosomal protein S17 | 802.28 | + | |
| PADG_00692 | elongation factor 1-alpha | 7568.61 | 0.29 | |
| PADG_06265 | elongation factor 1 gamma domain-containing protein | 5531 | 0.57 | |
| PADG_08125 | elongation factor 2 | 3952.23 | 0.48 | |
| PADG_07105 | arginine-tRNA ligase | 554.11 | + | |
| PADG_08472 | lysine-tRNA ligase | 503.01 | + | |
| **PROTEIN FOLDING, MODIFICATION, DESTINATION** | |  |  | |
| PADG_07515 | UBX domain-containing protein | 573.32 | 1.49 | |
| PADG_01852 | small glutamine-rich tetratricopeptide repeat-containing protein | 991 | 2.2 | |
| PADG_04034 | chaperone DnaJ | 924.8 | 1.5 | |
| PADG_02637 | ubiquitin-conjugating enzyme | 795.87 | *** | |
| PADG_01605 | polyubiquitin | 2988.76 | 2.24 | |
| PADG_03424 | ubiquitin-activating enzyme E1 | 1565.4 | 1.52 | |
| PADG_07558 | ubiquitin carboxyl-terminal hydrolase | 735.58 | 1.49 | |
| PADG_07715 | hsp90-like protein | 21307.69 | 0.35 | |
| PADG_08369 | hsp60-like protein | 31486.72 | 0.69 | |
| **PROTEIN DEGRADATION** | |  |  | |
| PADG_00599 | 26S protease regulatory subunit 6A | 436.54 | *** | |
| PADG_01935 | 26S proteasome non-ATPase regulatory subunit 10 | 726.98 | 1.5 | |
| PADG_03192 | proteasome component PUP2 | 484.74 | 1.59 | |
| PADG_03221 | thimet oligopeptidase | 769.99 | 2.03 | |
| PADG_03735 | proline iminopeptidase | 574.84 | *** | |
| PADG_03967 | proteasome component C5 | 3676.41 | 1.44 | |
| PADG_04952 | AAA ATPase | 491.31 | *** | |
| PADG_05160 | dipeptidyl-peptidase | 435.87 | 1.87 | |
| PADG_04167 | aspartyl aminopeptidase | 456.35 | 1.43 | |
| PADG_05193 | xaa-Pro aminopeptidase | 465.6 | *** | |
| PADG_05560 | 26S proteasome regulatory subunit rpn-1 | 757.72 | *** | |
| PADG_05820 | xaa-Pro aminopeptidase | 606.66 | 1.59 | |
| PADG_06766 | mitochondrial-processing peptidase subunit beta | 944.78 | 3.18 | |
| PADG_05837 | E3 ubiquitin ligase complex SCF subunit sconC | 3142.06 | 1.55 | |
| PADG_03727 | proteasome component PUP1 | 2198.59 | 0.67 | |
| **PROTEIN BINDING** | |  |  | |
| PADG_07884 | polyadenylate-binding protein, cytoplasmic and nuclear | 459.2 | 2.63 | |
| PADG_07249 | actin binding protein | 460.77 | 1.99 | |
| PADG_01529 | TPR repeat protein | 640.89 | *** | |
| **CELLULAR TRANSPORT** | |  |  | |
| PADG_03203 | BAR domain-containing protein | 1831.27 | 1.71 | |
| PADG_08188 | vacuolar-sorting protein snf7 | 895.93 | *** | |
| PADG_02833 | ADP-ribosylation fator | 2358.28 | + | |
| **CELL RESCUE, DEFENSE AND VIRULENCE** | |  |  | |
| PADG_01551 | thioredoxin reductase | 976.26 | 1.56 | |
| PADG_01954 | superoxide dismutase 2 Fe-Mn | 1379.02 | 1.99 | |
| PADG_07418 | superoxide dismutase 1 Cu-Zn | 2464.72 | 1.99 | |
| PADG_02030 | Hsp90 co-chaperone Cdc37 | 2932.07 | 2.22 | |
| PADG_02785 | heat shock protein Hsp88 | 9518.65 | 1.5 | |
| PADG_03963 | 30 kDa heat shock protein | 6432.9 | 1.84 | |
| PADG_04379 | heat shock protein STI1 | 4552.74 | 1.99 | |
| PADG_01479 | gamma-glutamyltransferase | 545.4 | 1.76 | |
| PADG_07422 | serine proteinase | 380.41 | 1.8 | |
| PADG_07460 | vacuolar aminopeptidase | 487.7 | 1.53 | |
| PADG_00324 | catalase P | 1207.99 | 0.60 | |
| PADG_03095 | mitochondrial peroxiredoxin PRX1 | 2391.55 | 0.67 | |
| PADG_03163 | cytochrome c peroxidase mitochondrial | 7277.58 | 0.47 | |
| PADG_08651 | peroxisomal hydratase-dehydrogenase-epimerase | 724.34 | + | |
| PADG_07946 | peroxisomal matrix protein | 4560.11 | 0.6 | |
| PADG_02048 | nitroreductase family protein | 636.86 | + | |
| **CELL DEATH** | |  |  | |
| PADG_06087 | programmed cell death protein 5 | 578.79 | 2.22 | |
| **UNCLASSIFIED** | |  |  | |
| PADG_06289 | hypothetical protein | 749.22 | 1.4 | |
| PADG_03869 | hypothetical protein | 785.98 | *** | |
| PADG_03827 | hypothetical protein | 2531.19 | *** | |
| PADG_01010 | hypothetical protein | 690.72 | 2.20 | |
| PADG_01871 | hypothetical protein | 725.03 | *** | |
| PADG_03210 | hypothetical protein | 1477.99 | 1.85 | |
| PADG_00496 | hypothetical protein | 515.07 | *** | |
| PADG_08480 | hypothetical protein | 646.53 | 3.85 | |
| PADG_11936 | hypothetical protein | 1140.44 | *** | |
| PADG_02307 | hypothetical protein | 740.98 | *** | |
| PADG_04442 | hypothetical protein | 452.53 | *** | |
| PADG_04229 | hypothetical protein | 583.82 | 1.56 | |
| PADG_07627 | 4-carboxymuconolactone decarboxylase family protein | 628.84 | 1.93 | |
| PADG_12437 | EF hand domain-containing protein | 391.93 | *** | |
| PADG_05837 | E3 ubiquitin ligase complex SCF subunit sconC | 3142.06 | 1.55 | |
| PADG_03526 | M protein repeat protein | 467.91 | *** | |
| PADG_00344 | hypothetical protein | 1008.02 | + | |
| PADG_00211 | hypothetical protein | 2047.72 | + | |
| PADG_00921 | hypothetical protein | 2142.99 | 0.63 | |
| PADG_01488 | hypothetical protein | 1980.81 | + | |
| PADG_03660 | hypothetical protein | 860.43 | + | |
| PADG_02764 | hypothetical protein | 2170.39 | 0.71 | |
| PADG_08212 | hypothetical protein | 2557.19 | 0.26 | |
| PADG_08368 | hypothetical protein | 716.93 | 0.34 | |
| PADG_01849 | GTP-binding protein YchF | 416.64 | + | |
| PADG_07287 | WD repeat-containing protein | 579.21 | + | |
| PADG_08483 | chromobox protein 1 | 411.76 | 0.58 | |
| PADG_07412 | DUF1479 domain-containing protein | 861.11 | + | |
| PADG_05356 | isochorismatase domain-containing protein | 871.15 | 0.55 | |
| PADG_04559 | progesterone binding protein | 1333.07 | + | |
| PADG_06196 | 12-oxophytodienoate reductase | 2364.81 | 0.63 | |

^1^Accession number obtained in the *Paracoccidioides* database available at <http://www.broadinstitute.org/annotation/genome/paracoccidioides_brasiliensis/MultiHome.html>.

^2^PLGS score is the result of different mathematical models for peptide and fragment assign prediction. Acceptable score values consider protein identification with a minimum confidence level of 95% and a false discovery rate of 6%.

^3^Ratio values were obtained by dividing the values of protein abundance (in fmol) from *Pb*18 during infection of activated macrophages by the abundance in control**.** Proteins with a minimum fold change of 40% were considered regulated.

^4^Biological process of differentially expressed proteins from MIPS (http://mips.helmholtz-muenchen.de/funcatDB/) and Uniprot databases (http://www.uniprot.org/).

*** Proteins detected in *P. brasiliensis* *Pb*18 only during activated macrophage infection.

+ Proteins detected in *P. brasiliensis* *Pb*18 only in the control condition.
