# Supplementary material for: Proteomic Analysis of Paracoccidioides brasiliensis During Infection of Alveolar Macrophages Primed or Not by Interferon-Gamma
Source: Front Microbiol. 2019 Feb 5;10:96. doi: 10.3389/fmicb.2019.00096 (PMC6371752; doi:10.3389/fmicb.2019.00096)
Supplement: Supplementary file 5 [file Table_3.docx]

**Supplementary Table 3. Regulated proteins of *P. brasiliensis*, *Pb*18 after 6 h infection in non-activated alveolar macrophages**

| **Accession number^1^** | **Protein description** | **Score^2^** | ***Pb*18_NP x CTRL**  **Ratio^3^** |
| --- | --- | --- | --- |
| **Functional categories^4^** | |  |  |
| **AMINO ACID METABOLISM** | |  |  |
| **Amino acid degradation** | |  |  |
| PADG_00822 | glutaminase A | 439.09 | *** |
| PADG_04516 | NADP-specific glutamate dehydrogenase | 1576.71 | 1.90 |
| PADG_06429 | ketol-acid reductoisomerase, mitochondrial | 1960.68 | 1.82 |
| PADG_05922 | glutamate carboxypeptidase | 5325.3 | 2.09 |
| PADG_05085 | 1-pyrroline-5-carboxylate dehydrogenase | 1535.16 | 1.62 |
| PADG_06546 | puromycin-sensitive aminopeptidase | 739.86 | 1.43 |
| PADG_03020 | alanine-glyoxylate aminotransferase | 565.67 | 1.98 |
| PADG_08465 | fumarylacetoacetase | 718.43 | 1.48 |
| PADG_03671 | phenylpyruvate tautomerase | 789.12 | 1.5 |
| PADG_01963 | glycine cleavage system H protein | 1151.78 | + |
| PADG_02214 | 4-aminobutyrate aminotransferase | 1496.37 | 0.68 |
| PADG_04570 | branched-chain amino acid aminotransferase | 979.87 | 0.7 |
| PADG_03514 | 2-oxoisovalerate dehydrogenase subunit alpha. mitochondrial | 761.58 | + |
| PADG_03466 | 3-hydroxyisobutyrate dehydrogenase | 504.5 | + |
| PADG_01621 | aspartate aminotransferase mitochondrial | 710.05 | + |
| PADG_03686 | aspartate aminotransferase cytoplasmic | 395.76 | + |
| PADG_00832 | adenylosuccinate synthetase | 969.84 | + |
| PADG_00215 | aromatic-L-amino-acid decarboxylase | 579.63 | + |
| PADG_05337 | glutamate-5-semialdehyde dehydrogenase | 487.9 | + |
| **Amino acid biosynthesis** | |  |  |
| PADG_06382 | acetyl-CoA acetyltransferase | 3416.52 | 1.65 |
| PADG_04689 | N-acetyl-gamma-glutamyl-phosphate reductase | 532.7 | *** |
| PADG_00602 | type I protein arginine methyltransferase | 1103.03 | 1.8 |
| PADG_01615 | homocitrate synthase, mitochondrial | 2141.48 | 2.39 |
| PADG_08376 | aspartate-semialdehyde dehydrogenase | 2633.05 | 2.37 |
| PADG_02914 | glycine cleavage system T protein | 1013.86 | 1.51 |
| PADG_07609 | dihydroxy-acid dehydratase | 1382.4 | 2.73 |
| PADG_07907 | acetolactate synthase | 2642.52 | 2.29 |
| PADG_00663 | homoserine dehydrogenase | 898.23 | 1.51 |
| PADG_04522 | homoserine kinase | 1291.2 | *** |
| PADG_02456 | cystathionine gamma-lyase | 645.01 | 1.47 |
| PADG_05111 | serine hydroxymethyltransferase, cytosolic | 2403.18 | 2.44 |
| PADG_05277 | serine hydroxymethyltransferase | 6324.08 | 1.64 |
| PADG_00210 | glycine dehydrogenase | 3064.66 | 1.56 |
| PADG_00402 | betaine aldehyde dehydrogenase | 453.52 | 1.45 |
| PADG_08406 | O-acetylhomoserine (thiol)-lyase | 16850.46 | 2.01 |
| PADG_03522 | methylthioadenosine phosphorylase | 1437.78 | 3.01 |
| PADG_08328 | 5-methyltetrahydropteroyltriglutamate-homocysteine S-methyltransferase | 9967.91 | 2.37 |
| PADG_08662 | cystathionine beta-lyase | 345.82 | *** |
| PADG_02726 | cysteine synthase | 1818.8 | 1.76 |
| PADG_01928 | S-adenosylmethionine synthase | 1737.8 | 1.47 |
| PADG_07274 | anthranilate synthase component 2 | 730.39 | 1.68 |
| PADG_01530 | guanine nucleotide-binding protein subunit beta-like protein | 11353.25 | 1.48 |
| PADG_06740 | betaine aldehyde dehydrogenase | 1292.59 | 0.75 |
| PADG_01418 | cysteine dioxygenase | 767.22 | + |
| PADG_04193 | cystathionine beta-synthase | 532.75 | + |
| **UREA CYCLE** |  |  |  |
| PADG_01305 | argininosuccinate lyase | 551.69 | *** |
| PADG_00637 | Arginase | 463.97 | 0.68 |
| PADG_00888 | argininosuccinate synthase | 652.7 | + |
| **NITROGEN METABOLISM** | |  |  |
| PADG_00446 | oxidoreductase 2-nitropropane dioxygenase family | 1406.89 | 1.56 |
| PADG_06490 | formamidase | 4731.69 | 2.02 |
| **NUCLEOTIDE/NUCLEOSIDE/NUCLEOBASE METABOLISM** | |  |  |
| PADG_02183 | ADP-ribose pyrophosphatase | 1018.86 | 1.72 |
| PADG_02246 | adenosine kinase | 1023.67 | 1.75 |
| PADG_04828 | adenylosuccinate lyase | 1267.14 | 1.97 |
| PADG_07585 | inosine-5'-monophosphate dehydrogenase | 1285.04 | 1.97 |
| PADG_01100 | uracil phosphoribosyltransferase | 1806.58 | + |
| **C-COMPOUND AND CARBOHYDRATE METABOLISM** | |  |  |
| PADG_11132 | phosphoglucomutase | 1205.72 | 1.61 |
| PADG_00298 | FGGY-family carbohydrate kinase | 573.38 | *** |
| PADG_03278 | inositol-3-phosphate synthase | 687.16 | *** |
| PADG_04374 | UTP-glucose-1-phosphate uridylyltransferase | 934.38 | *** |
| PADG_07435 | sorbitol utilization protein SOU2 | 3257.33 | 1.76 |
| PADG_03943 | phosphomannomutase | 736.23 | *** |
| PADG_00912 | UDP-galactopyranose mutase | 1876.92 | 2.46 |
| PADG_04312 | UDP-N-acetylglucosamine pyrophosphorylase | 435.09 | 1.4 |
| PADG_07523 | neutral alpha-glucosidase AB | 2916.74 | 2.54 |
| PADG_04761 | mannosyl-oligosaccharide glucosidase | 1036.62 | 2.25 |
| PADG_04148 | alpha-mannosidase | 1272.3 | 1.7 |
| PADG_12426 | 1,4-alpha-glucan-branching enzyme | 908.18 | 1.86 |
| **PENTOSE-PHOSPHATE PATHWAY** | |  |  |
| PADG_04604 | transketolase | 1424.25 | 1.56 |
| PADG_07771 | 6-phosphogluconolactonase | 619.03 | 1.43 |
| PADG_03651 | 6-phosphogluconate dehydrogenase, decarboxylating 1 | 1094.46 | 2.41 |
| PADG_07420 | transaldolase | 1467.82 | + |
| **GLYCOLYSIS/GLUCONEOGENESIS** | |  |  |
| PADG_03813 | hexokinase | 1973.12 | 1.44 |
| PADG_07950 | glucokinase | 622.95 | 1.87 |
| PADG_00451 | glucose-6-phosphate isomerase | 526.87 | 1.77 |
| PADG_05109 | 2,3-bisphosphoglycerate-independent phosphoglycerate mutase | 709.59 | 1.63 |
| PADG_04059 | Enolase | 29089.92 | 1.53 |
| PADG_01278 | pyruvate kinase | 1317.95 | 1.43 |
| PADG_08503 | phosphoenolpyruvate carboxykinase | 1337.49 | 1.63 |
| PADG_01896 | phosphoglycerate kinase | 2190.5 | 0.66 |
| **PYRUVATE METABOLISM** | |  |  |
| PADG_01797 | pyruvate dehydrogenase protein X componente | 536.58 | 1.52 |
| PADG_04165 | pyruvate dehydrogenase complex component Pdx1 | 4691.21 | 2.12 |
| **TRICARBOXYLIC ACID CYCLE** | |  |  |
| PADG_08387 | citrate synthase mitochondrial | 567.91 | 1.46 |
| PADG_11845 | aconitate hydratase mitochondrial | 1337.16 | 1.65 |
| PADG_00317 | succinyl-CoA ligase subunit beta | 1165.76 | 2.65 |
| PADG_04939 | succinyl-CoA:3-ketoacid-coenzyme A transferase subunit B | 1187.44 | 1.63 |
| PADG_01762 | oxoglutarate dehydrogenase (succinyl-transferring), E1 component | 1134.35 | 1.59 |
| PADG_00052 | succinate dehydrogenase [ubiquinone] flavoprotein subunit, mitochondrial | 2588.78 | 1.81 |
| PADG_08119 | fumarate hydratase, mitochondrial | 949.77 | 2.25 |
| PADG_08054 | malate dehydrogenase, NAD-dependent | 6213.22 | 1.46 |
| PADG_04994 | ATP-citrate-lyase | 503.4 | 0.65 |
| **METHYLCITRATE CYCLE** | |  |  |
| PADG_04710 | 2-methylcitrate synthase, mitochondrial | 4668.51 | 1.95 |
| PADG_04718 | 2-methylcitrate dehydratase | 13900.44 | 1.53 |
| PADG_04709 | methyl-isocitrate lyase | 1366.54 | 1.55 |
| **GLYOXALATE CYCLE** | |  |  |
| PADG_01483 | isocitrate lyase | 782.07 | 1.59 |
| **ELECTRON TRANSPORT AND RESPIRATION** | |  |  |
| PADG_07749 | NAD(P)H:quinone oxidoreductase, type IV | 63456.09 | 2.34 |
| PADG_01366 | NADH-ubiquinone oxidoreductase 1  alpha subcomplex subunit 5 | 8527.01 | 2.21 |
| PADG_02745 | NADH-ubiquinone oxidoreductase Fe-S protein 6 | 3903.78 | *** |
| PADG_06978 | cytochrome C | 2091.97 | 1.68 |
| PADG_04397 | cytochrome c oxidase subunit 4, mitochondrial | 16957.84 | 3.55 |
| PADG_05750 | putative cytochrome c oxidase subunit Via | 40028.16 | 3.89 |
| PADG_07081 | electron transfer flavoprotein subunit alpha | 1912.68 | 1.74 |
| PADG_11468 | electron transfer flavoprotein beta-subunit | 457.76 | *** |
| PADG_11981 | V-type proton ATPase catalytic subunit A | 452.37 | 1.81 |
| PADG_04319 | V-type ATPase, G subunit | 1496.06 | 2.08 |
| PADG_03175 | V-type proton ATPase subunit F | 5509.99 | 2.88 |
| PADG_00688 | F-type H+-transporting ATPase subunit H | 1411.4 | 2.56 |
| PADG_08391 | plasma membrane ATPase | 1062.2 | 1.41 |
| PADG_08394 | cytochrome b-c1 complex subunit 2 | 904.65 | + |
| PADG_06221 | formate dehydrogenase | 632.87 | + |
| **ATP SYNTHESIS** | |  |  |
| PADG_07042 | ATP synthase F1, delta subunit | 2655.74 | *** |
| PADG_07813 | ATP synthase F1, gamma subunit | 5080.46 | 2.02 |
| PADG_04729 | ATP synthase subunit D, mitochondrial | 10759.93 | 3.24 |
| PADG_08349 | ATP synthase subunit beta, mitochondrial | 44454.03 | 2.92 |
| PADG_07789 | ATP synthase subunit delta, mitochondrial | 18673.08 | 2.53 |
| PADG_07964 | vacuolar ATP synthase subunit E | 866.17 | 1.52 |
| **FATTY ACID BIOSYNTHESIS** | |  |  |
| PADG_00254 | fatty acid synthase subunit alpha | 524.97 | 1.62 |
| PADG_00255 | fatty acid synthase subunit beta dehydratase | 1306.32 | 2.15 |
| PADG_05310 | leukotriene A-4 hydrolase | 2329.58 | 2.17 |
| PADG_00608 | formyl-coenzyme A transferase | 457.47 | 1.93 |
| PADG_05783 | farnesyl pyrophosphate synthetase | 752.27 | *** |
| **OXIDATION OF FATTY ACIDS** | |  |  |
| PADG_01486 | short chain dehydrogenase/reductase family | 1481.15 | + |
| PADG_01209 | enoyl-CoA hydratase | 4337.27 | 0.61 |
| PADG_03194 | 3-ketoacyl-CoA thiolase B | 906.43 | 0.5 |
| PADG_03449 | isopentenyl-diphosphate delta-isomerase | 795.96 | + |
| PADG_04343 | short chain dehydrogenase/reductase | 620.43 | + |
| **METABOLISM OF VITAMINS. COFACTORS. AND PROSTHETIC GROUPS** | |  |  |
| PADG_00443 | dihydropteroate synthase | 2037.91 | 1.55 |
| PADG_01886 | adenosylhomocysteinase | 5114.94 | 2.08 |
| PADG_04032 | uroporphyrinogen decarboxylase | 562.49 | *** |
| PADG_05947 | nicotinate-nucleotide diphosphorylase (carboxylating) | 11884.09 | 1.72 |
| PADG_00513 | 2-succinylbenzoate-CoA ligase | 484.5 | *** |
| **SECONDARY METABOLISM** | |  |  |
| PADG_04175 | inorganic pyrophosphatase | 3036.59 | 1.62 |
| PADG_04899 | metallo-beta-lactamase domain-containing protein | 614.27 | *** |
| PADG_04603 | spermidine synthase | 7353.23 | 1.45 |
| PADG_08108 | coproporphyrinogen III oxidase | 714.31 | *** |
| PADG_04636 | dienelactone hydrolase family protein | 1618.93 | 1.56 |
| PADG_08034 | dienelactone hydrolase family protein | 1892.29 | 2.09 |
| **SIGNAL TRANSDUCTION** | |  |  |
| PADG_02017 | calmodulin | 6723.5 | 2.7 |
| PADG_01565 | calnexin | 1281.42 | 1.72 |
| PADG_06273 | calcineurin subunit B | 845.86 | *** |
| PADG_04440 | 14-3-3-like protein 2 | 23558.55 | 1.68 |
| PADG_04056 | 14-3-3 family protein épsilon | 18174.25 | 1.9 |
| PADG_11275 | CMGC/MAPK protein kinase | 439.81 | + |
| PADG_00172 | ras-like GTP-binding protein | 506.63 | + |
| **CYTOSKELETON** | |  |  |
| PADG_00128 | tubulin alpha-2 chain | 1787.02 | 1.45 |
| PADG_05239 | tubulin-specific chaperone Rbl2 | 1188.96 | 1.70 |
| PADG_00422 | actin cytoskeleton protein | 12255.21 | 2.37 |
| PADG_02157 | actin cytoskeleton-regulatory complex protein END | 636.1 | *** |
| PADG_12076 | actin beta/gamma 1 | 2871.4 | 1.71 |
| PADG_05538 | actin | 2068.43 | 2.82 |
| PADG_03219 | myosin regulatory light chain cdc4 | 2427.08 | 1.9 |
| PADG_08615 | Tropomyosin | 681.04 | 1.47 |
| **CELL CYCLE** | |  |  |
| PADG_12437 | EF hand domain-containing protein | 604.69 | *** |
| PADG_03073 | nuclear movement protein nudC | 8082.11 | 1.76 |
| PADG_00849 | nuclear segregation protein Bfr1 | 1157.96 | 1.64 |
| PADG_05683 | cell division control protein 48 | 906.88 | 1.76 |
| PADG_11679 | proliferating cell nuclear antigen (pcna) | 4415.85 | 1.95 |
| PADG_02763 | cyclin-dependent kinase regulatory subunit | 763.95 | + |
| **DNA PROCESSING** | |  |  |
| PADG_05798 | single-strand binding protein family | 27710.18 | 1.61 |
| PADG_02683 | UV excision repair protein Rad23 | 8139.56 | 2.37 |
| PADG_00656 | non-histone chromosomal protein 6 | 2992.07 | 2.11 |
| PADG_00615 | proteasome component C7-alpha | 3316.5 | 1.61 |
| PADG_05906 | histone H2A | 1859.72 | 1.93 |
| PADG_05907 | histone H2B | 18240.2 | 1.68 |
| PADG_00718 | histone chaperone asf1 | 2760.63 | 1.76 |
| PADG_00872 | histone | 18101.32 | 2.78 |
| PADG_00873 | histone H3 | 4340.91 | 2.71 |
| PADG_07134 | histone H4 | 17501.65 | 3.53 |
| **TRANSCRIPTION** | |  |  |
| PADG_07888 | eukaryotic translation initiation factor 5ª | 628.13 | *** |
| PADG_11711 | ATP-dependent RNA helicase eIF4A | 1321.54 | 2.67 |
| PADG_05034 | RNA binding domain-containing protein | 867.11 | 1.54 |
| PADG_01455 | KH domain RNA-binding protein | 409.15 | 1.59 |
| PADG_06180 | cap binding protein | 582.21 | 1.62 |
| PADG_00067 | polymerase II polypeptide D | 3632.22 | *** |
| PADG_01151 | DNA-directed RNA polymerase II subunit RPB11 | 551.42 | *** |
| PADG_04657 | nascent polypeptide-associated complex subunit beta | 5769.23 | 3.78 |
| PADG_04730 | nascent polypeptide-associated complex subunit alpha | 18126.22 | 1.87 |
| PADG_04966 | DNA-directed RNA polymerase II subunit RPB11 | 728.43 | 1.72 |
| PADG_02825 | small nuclear ribonucleoprotein Lsm8 | 1929.44 | 2.26 |
| PADG_03696 | nuclear polyadenylated RNA-binding protein Nab2 | 1527.97 | *** |
| PADG_05393 | mRNA decapping hydrolase | 1092.36 | 3.20 |
| PADG_04672 | ATP-dependent RNA helicase SUB2 | 697.62 | *** |
| PADG_04796 | pre-mRNA-splicing factor rse1 | 445.01 | *** |
| PADG_05587 | U2 small nuclear ribonucleoprotein B | 617.42 | 1.44 |
| PADG_06734 | U6 snRNA-associated Sm-like protein LSm7 | 2246.48 | *** |
| PADG_11424 | cleavage and polyadenylation specific subunit | 550.08 | 1.56 |
| PADG_11958 | small nuclear ribonucleoprotein | 2502.26 | *** |
| PADG_00676 | rnapii degradation factor def1 | 1208.35 | 2.53 |
| PADG_07469 | RNase III domain-containing protein | 797.44 | 1.93 |
| PADG_04981 | nucleoporin p58/p45 | 531.32 | *** |
| PADG_11062 | bZIP transcription fator | 6178.35 | 2.78 |
| PADG_07416 | transcription factor | 474.55 | + |
| PADG_06768 | rRNA 2'-O-methyltransferase fibrillarin | 836.55 | + |
| **PROTEIN SYNTHESIS** | |  |  |
| PADG_07515 | UBX domain-containing protein | 614.94 | 1.95 |
| PADG_04057 | translation initiation factor 3 subunit J | 2426.37 | 1.9 |
| PADG_02759 | ribosome recycling fator | 3731.33 | 3.75 |
| PADG_05939 | 60S ribosomal protein L27a | 2405.32 | 1.4 |
| PADG_00995 | ubiquitin-40S ribosomal protein S27a | 1131.09 | 1.75 |
| PADG_01427 | 40S ribosomal protein S12 | 3942.19 | 1.69 |
| PADG_02056 | ribosomal protein L7/L12 | 9257.25 | 1.76 |
| PADG_02446 | 60S acidic ribosomal protein P2 | 17861.02 | 4.56 |
| PADG_04866 | 40S ribosomal protein S10-A | 1188.82 | 1.5 |
| PADG_06680 | 40S ribosomal protein S22 | 6671.03 | 2.29 |
| PADG_01949 | translation elongation factor Tu | 8904.08 | 1.97 |
| PADG_04034 | chaperone DnaJ | 2904.89 | 1.54 |
| PADG_00207 | DnaJ like subfamily C member 2 | 793.49 | 1.42 |
| PADG_02206 | DnaJ domain protein Psi | 613.07 | 2.07 |
| PADG_01079 | translation initiation factor 4B | 1039.02 | 1.76 |
| PADG_02691 | eukaryotic translation initiation factor 6 | 2002.04 | 1.61 |
| PADG_08033 | eukaryotic translation initiation factor 3 subunit B | 489.21 | 1.97 |
| PADG_01558 | histidyl-tRNA synthetase | 556.29 | 2.63 |
| PADG_00514 | 60S ribosomal protein L16 | 4223.18 | + |
| PADG_07924 | 60S ribosomal protein L24 | 433.47 | + |
| PADG_01083 | 60S ribosomal protein L32 | 649.58 | 0.61 |
| PADG_01267 | 40S ribosomal protein S11 | 7018.59 | 0.48 |
| PADG_01281 | mitochondrial 37S ribosomal protein MRPS8 | 463.22 | + |
| PADG_02797 | mitochondrial 54S ribosomal protein YmL3 | 428.68 | + |
| PADG_04449 | 60S ribosomal protein L23 | 1563.11 | + |
| PADG_05338 | 60S ribosomal protein L18-B | 7862.46 | 0.52 |
| PADG_06875 | 50S ribosomal protein L4 | 417.91 | + |
| PADG_00355 | 40S ribosomal protein S17 | 802.28 | + |
| PADG_08213 | ribosomal protein S2 | 593.74 | + |
| PADG_07105 | arginine-tRNA ligase | 554.11 | + |
| PADG_08472 | lysine-tRNA ligase | 503.01 | + |
| **PROTEIN FOLDING, MODIFICATION, DESTINATION** | |  |  |
| PADG_01605 | polyubiquitin | 1112.16 | 1.4 |
| PADG_01852 | small glutamine-rich tetratricopeptide repeat-containing protein | 1537.32 | 2.49 |
| PADG_05183 | Grx4 family monothiol glutaredoxin | 1644.83 | 2.12 |
| PADG_12323 | peptidyl-prolyl cis-trans isomerase | 804.14 | 1.85 |
| PADG_06488 | peptidyl-prolyl cis-trans isomerase D | 19095.81 | 1.42 |
| PADG_06992 | mitochondrial co-chaperone GrpE | 8275.4 | 1.76 |
| PADG_05032 | Hsp90 binding co-chaperone (Sba1) | 4917.89 | 1.59 |
| PADG_03424 | ubiquitin-activating enzyme E1 | 1345.75 | 1.72 |
| PADG_07558 | ubiquitin carboxyl-terminal hydrolase | 523.42 | 1.55 |
| PADG_08270 | UBX domain-containing protein | 493.3 | *** |
| PADG_00430 | hsp7-like protein | 5500.47 | 0.59 |
| **PROTEIN DEGRADATION** | |  |  |
| PADG_06766 | mitochondrial-processing peptidase subunit beta | 3201 | 2.31 |
| PADG_03221 | thimet oligopeptidase | 1073.19 | 2.08 |
| PADG_04167 | aspartyl aminopeptidase | 1162.18 | 1.7 |
| PADG_03735 | proline aminopeptidase | 422.42 | *** |
| PADG_05160 | dipeptidyl-peptidase | 567.55 | 2.29 |
| PADG_00599 | 26S protease regulatory subunit 6A | 602.27 | *** |
| PADG_02735 | proteasome component PRE6 | 5654.91 | 1.59 |
| PADG_03192 | proteasome component PUP2 | 713.27 | 2.36 |
| PADG_04952 | AAA ATPase | 571.94 | *** |
| PADG_05193 | xaa-Pro aminopeptidase PEPP | 1126.68 | *** |
| PADG_05820 | xaa-Pro aminopeptidase P | 1428.19 | 1.91 |
| PADG_03680 | proteasome component PRE2 | 1506.31 | 1.61 |
| PADG_03965 | proteasome component PRE4 | 2323.35 | 1.80 |
| PADG_03967 | proteasome component C5 | 9826.24 | 1.62 |
| PADG_03982 | proteasome component C1 | 3157.46 | 1.75 |
| PADG_04067 | proteasome component PUP3 | 1678.89 | 1.63 |
| PADG_07190 | proteasome component Y7 | 811.6 | 1.61 |
| PADG_08087 | proteasome component PRE3 | 5539.32 | 1.53 |
| PADG_00634 | vacuolar protease A | 329.52 | *** |
| **PROTEIN/NUCLEOTIDE/METAL BINDING** | |  |  |
| PADG_04934 | RNP domain protein | 619.2 | 1.63 |
| PADG_07249 | actin binding protein | 989.85 | 2.34 |
| PADG_01032 | DNA-binding protein, 42 kDa | 1140.68 | *** |
| PADG_02652 | Grp1p | 36483.8 | 1.71 |
| PADG_04311 | cellular nucleic acid-binding protein | 1688.29 | 1.91 |
| PADG_07884 | polyadenylate-binding protein, cytoplasmic and nuclear | 490.6 | 1.8 |
| PADG_01529 | TPR repeat protein | 645.78 | *** |
| **CELLULAR TRANSPORT** | |  |  |
| PADG_07023 | carnitine O-acetyltransferase | 1083.11 | 1.45 |
| PADG_02022 | clathrin light chain | 3539.13 | 1.79 |
| PADG_06165 | glycolipid transferprotein HET-C2 | 883.23 | 2.29 |
| PADG_01994 | mitochondrial import receptor subunit tom22 | 1001.71 | *** |
| PADG_03274 | mitochondrial import inner membrane translocase subunit tim9 | 3109.89 | *** |
| PADG_02352 | copper chaperone | 2443.46 | *** |
| PADG_11950 | Ran-specific GTPase-activating protein 1 | 3845.45 | 1.84 |
| PADG_03203 | BAR domain-containing protein | 6023.64 | 2.73 |
| PADG_08188 | vacuolar-sorting protein snf7 | 1229.57 | *** |
| PADG_00622 | ATPase GET3 | 514.77 | + |
| **CELL RESCUE, DEFENSE AND VIRULENCE** | |  |  |
| PADG_01551 | thioredoxin reductase | 1081.7 | 1.84 |
| PADG_01954 | superoxide dismutase 2 Fe-Mn | 2543.95 | 4.72 |
| PADG_07418 | superoxide dismutase 1 Cu-Zn | 13608.78 | 2.78 |
| PADG_00778 | Hsp70 | 482.87 | 1.43 |
| PADG_02030 | Hsp90 co-chaperone Cdc37 | 4849.77 | 2.71 |
| PADG_02785 | heat shock protein Hsp88 | 6273.21 | 1.48 |
| PADG_03963 | 30 kDa heat shock protein | 15324.12 | 2.05 |
| PADG_04379 | heat shock protein STI1 | 11812.75 | 2.09 |
| PADG_01479 | gamma-glutamyltransferase | 1862.55 | 2.46 |
| PADG_06314 | carboxypeptidase Y | 924.76 | 1.97 |
| PADG_07422 | serine proteinase | 656.45 | 2.17 |
| PADG_07460 | vacuolar aminopeptidase | 1941.91 | 1.82 |
| PADG_07674 | carbonic anhydrase | 2074.37 | 1.55 |
| PADG_07946 | peroxisomal matrix protein | 2670.46 | 0.35 |
| PADG_08651 | peroxisomal hydratase-dehydrogenase-epimerase | 724.34 | + |
| **SECONDARY METABOLISM** | |  |  |
| PADG_01052 | 3-demethylubiquinone-9 3-methyltransferase | 1096.69 | + |
| **CELL DEATH** | |  |  |
| PADG_06336 | cell lysis protein cwl1 | 1040.98 | *** |
|  |  |  |  |
| **UNCLASSIFIED** | |  |  |
| PADG_00824 | hypothetical protein | 1685.96 | 1.52 |
| PADG_05474 | hypothetical protein | 1067.5 | 1.58 |
| PADG_03869 | hypothetical protein | 639.41 | *** |
| PADG_03788 | hypothetical protein | 833.59 | *** |
| PADG_05884 | hypothetical protein | 7917.75 | 2.28 |
| PADG_07064 | hypothetical protein | 1199.21 | 1.94 |
| PADG_04934 | hypothetical protein | 619.2 | 1.63 |
| PADG_00828 | hypothetical protein | 837.5 | 2.41 |
| PADG_11936 | hypothetical protein | 600.69 | *** |
| PADG_00496 | hypothetical protein | 941.2 | *** |
| PADG_03827 | hypothetical protein | 2950.62 | *** |
| PADG_01010 | hypothetical protein | 1473.23 | 2.96 |
| PADG_05703 | hypothetical protein | 1162.77 | 1.77 |
| PADG_08368 | hypothetical protein | 3283.04 | 2.11 |
| PADG_03210 | hypothetical protein | 2128.5 | 2.56 |
| PADG_08480 | hypothetical protein | 631.36 | 2.8 |
| PADG_00921 | hypothetical protein | 8475.1 | 1.87 |
| PADG_07670 | hypothetical protein | 841.85 | 1.79 |
| PADG_01867 | hypothetical protein | 453.9 | 1.51 |
| PADG_03176 | hypothetical protein | 520.17 | 1.49 |
| PADG_08436 | hypothetical protein | 1263.02 | *** |
| PADG_05556 | hypothetical protein | 1218.06 | *** |
| PADG_06547 | hypothetical protein | 1233.13 | *** |
| PADG_02307 | hypothetical protein | 473.88 | *** |
| PADG_02092 | hypothetical protein | 1528.71 | 1.89 |
| PADG_08666 | hypothetical protein | 1921.5 | *** |
| PADG_02967 | hypothetical protein | 5909.55 | 1.76 |
| PADG_04229 | hypothetical protein | 646.38 | 2.04 |
| PADG_04439 | hypothetical protein | 11250.78 | 1.41 |
| PADG_03827 | hypothetical protein | 2950.62 | *** |
| PADG_02343 | MYG1 protein | 2421.65 | 1.68 |
| PADG_00694 | ankyrin repeat protein | 1375.38 | 1.84 |
| PADG_00463 | DUF833 domain-containing protein | 625.24 | 1.61 |
| PADG_02845 | diploid state maintenance protein chpA | 2809.36 | 1.5 |
| PADG_07714 | NTF2 and RRM domain-containing protein | 1009.12 | 1.59 |
| PADG_06515 | suaprga1 | 28036.56 | 1.48 |
| PADG_03526 | M protein repeat protein | 694.82 | *** |
| PADG_06182 | WD repeat-containing protein | 1138.44 | 1.63 |
| PADG_01488 | hypothetical protein | 1980.81 | + |
| PADG_04475 | hypothetical protein | 1040.31 | + |
| PADG_07870 | hypothetical protein | 638.76 | + |
| PADG_00211 | hypothetical protein | 2047.72 | + |
| PADG_03631 | hypothetical protein | 1247.15 | + |
| PADG_03660 | hypothetical protein | 860.43 | + |
| PADG_08212 | hypothetical protein | 1071 | 0.59 |
| PADG_01849 | GTP-binding protein YchF | 416.64 | + |
| PADG_00282 | GTP-binding protein ypt2 | 2005.04 | + |
| PADG_07627 | 4-carboxymuconolactone decarboxylase family protein | 628.84 | + |
| PADG_08483 | chromobox protein 1 | 816.66 | 0.75 |
| PADG_07412 | DUF1479 domain-containing protein | 861.11 | + |
| PADG_05356 | isochorismatase domain-containing protein | 871.15 | + |
| PADG_07287 | WD repeat-containing protein | 579.21 | + |
| PADG_02719 | dTDP-4-dehydrorhamnose reductase | 513.83 | + |

^1^Accession number obtained in the *Paracoccidioides* database available at <http://www.broadinstitute.org/annotation/genome/paracoccidioides_brasiliensis/MultiHome.html>.

^2^PLGS score is the result of different mathematical models for peptide and fragment assign prediction. Acceptable score values consider protein identification with a minimum confidence level of 95% and a false discovery rate of 6%.

^3^Ratio values were obtained by dividing the values of protein abundance (in fmol) from *Pb*18 during infection of activated macrophages by the abundance in control**.** Proteins with a minimum fold change of 40% were considered regulated.

^4^Biological process of differentially expressed proteins from MIPS (http://mips.helmholtz-muenchen.de/funcatDB/) and Uniprot databases (http://www.uniprot.org/).

*** Proteins detected in *P. brasiliensis* *Pb*18 only in non activated macrophage infection.

+ Proteins detected in *P. brasiliensis* *Pb*18 only in the control condition.
